# Off-targetP ML: an open source machine learning framework for off-target panel safety assessment of small molecules

**DOI:** 10.1186/s13321-022-00603-w

**Published:** 2022-05-07

**Authors:** Doha Naga, Wolfgang Muster, Eunice Musvasva, Gerhard F. Ecker

**Affiliations:** 1 Roche Pharma Research & Early Development, Roche Innovation Center Basel, F. Hoffmann-La Roche Ltd., Basel, Switzerland; 2 Department of Pharmaceutical Sciences, University of Vienna, Vienna, Austria

**Keywords:** Drug discovery, Safety screening, Off-target panel, Class imbalance, Deep learning, Automated machine learning (AutoML), Ensembling methods

## Abstract

Unpredicted drug safety issues constitute the majority of failures in the pharmaceutical industry according to several studies. Some of these preclinical safety issues could be attributed to the non-selective binding of compounds to targets other than their intended therapeutic target, causing undesired adverse events. Consequently, pharmaceutical companies routinely run in-vitro safety screens to detect off-target activities prior to preclinical and clinical studies. Hereby we present an open source machine learning framework aiming at the prediction of our in-house 50 off-target panel activities for ~ 4000 compounds, directly from their structure. This framework is intended to guide chemists in the drug design process prior to synthesis and to accelerate drug discovery. We also present a set of ML approaches that require minimum programming experience for deployment. The workflow incorporates different ML approaches such as deep learning and automated machine learning. It also accommodates popular issues faced in bioactivity predictions, as data imbalance, inter-target duplicated measurements and duplicated public compound identifiers. Throughout the workflow development, we explore and compare the capability of Neural Networks and AutoML in constructing prediction models for fifty off-targets of different protein classes, different dataset sizes, and high-class imbalance. Outcomes from different methods are compared in terms of efficiency and efficacy. The most important challenges and factors impacting model construction and performance in addition to suggestions on how to overcome such challenges are also discussed.

**Supplementary Information:**

The online version contains supplementary material available at 10.1186/s13321-022-00603-w.

## Introduction

In-depth exploration of drug candidates’ attrition through analysis of public or individual in-house data has been an ongoing trend in recent years [1–4]. Preclinical toxicity has been identified as one of the major reasons behind the failures of candidates in the early phases of drug development [2, 5]. Big efforts have been exerted to minimize undesired effects attributed to one of the key contributors to non-clinical toxicities, poly-pharmacology [6–8]. Poly-pharmacology or off-target activity is the interaction of the drug with targets other than the intended therapeutic target. This interaction may lead to adverse reactions, which can be seen in pre-clinical animal studies and clinical trials. Adverse events might also manifest in later stages such as post-marketing, eventually leading to drug withdrawal from the market [9].

Consequently, pharmaceutical companies routinely run safety in-vitro assays in early stages of drug development where the lead compounds are screened against off-targets and thus assessing the promiscuity of these compounds. A minimal panel of the most significant off-targets (~ 44 targets) were presented in the work of Bowes et al., where the relation between their activation/inhibition and undesired physiological effect has been extensively described [10].

Our in-house off- target optimized panel (composed of 50 targets) was presented along with the target’s selection methodology and assay protocol by Bendels et al. [11]. These routinely conducted safety screens (dating back to 2004) have resulted in an enriched off-target interaction database. Exploiting this historical data towards the prediction of the off-target activities directly from the compounds’ chemical structure is one of the key objective of this work. Anticipating early toxicities of chemical structures through in silico models prior to their synthesis could be a useful guide to medicinal chemists. Additionally, using such models in lieu of in vitro assays could reduce in vitro testing and consequently accelerate drug discovery. Since choosing and implementing the appropriate computational method for the predictions is not a simple task due to the presence of many approaches that vary in algorithms, time consumption, availability, resources, and required skills, developing a completely automated workflow for off-target predictions (Off-targetP ML) is another important highlight of this study.

Deep learning is one of the approaches evaluated and implemented in this work. In recent years, it has demonstrated success in prediction of bioactivity data over other shallow approaches (e.g. Random Forest or Support Vector Machine), especially when applied to large datasets, and has ranked among the top performing method for QSAR predictions in the Kaggle and Tox21 challenge [12–14].

Another emerging approach in the field of ML is Automated Machine Learning (AutoML), which has recently shown a high impact in healthcare research and drug discovery [15]. The major difference between AutoML and other machine learning methods is the automatic feature selection and hyper parameter optimization, which are among the trickiest steps of building a ML model. With regard to our project, the 50 off-targets had varying active/inactive ratios and varying number of samples, and therefore different model architecture and hyper parameters tuning were required and should be accounted for, which is automatically established in AutoML tools. Furthermore, constructing a neural network often requires considerable computer science skills and experience, while several AutoML approaches might require little or no programming expertise and are available as a user interface (e.g. H2O driverless AI) or open-source packages, where the models can be executed through few command lines (e.g. AutoGluon Tabular and Auto-Sklearn). Another advantage of AutoML is the stacking of several models together to obtain an overall best accuracy. Comparing these three AutoML approaches to deep learning was therefore considered in this study for the sake of implementing the top performing method in the Off-targetP ML workflow.

The major challenge of this work is handling the data imbalance and understanding its impact on the model performance. The impact of other factors like target families and dataset sizes were also explored. Finally, through six case studies we test our workflow on chemogenomic publicly available datasets and demonstrated the effect of inhouse-public data enrichment in overcoming data imbalance.

## Methods

### The Off-targetP ML workflow

The two main purposes of this workflow are to construct new off-target models for any given off-target interaction dataset and to predict the off-target profile for any given chemical structure. Five machine learning approaches were adopted and compared in this work: Neural Networks, Random Forest, Auto-Sklearn, AutoGluon and H2O. The workflow implements the top performing method identified in this work: Neural Networks.

**Fig. 1:**
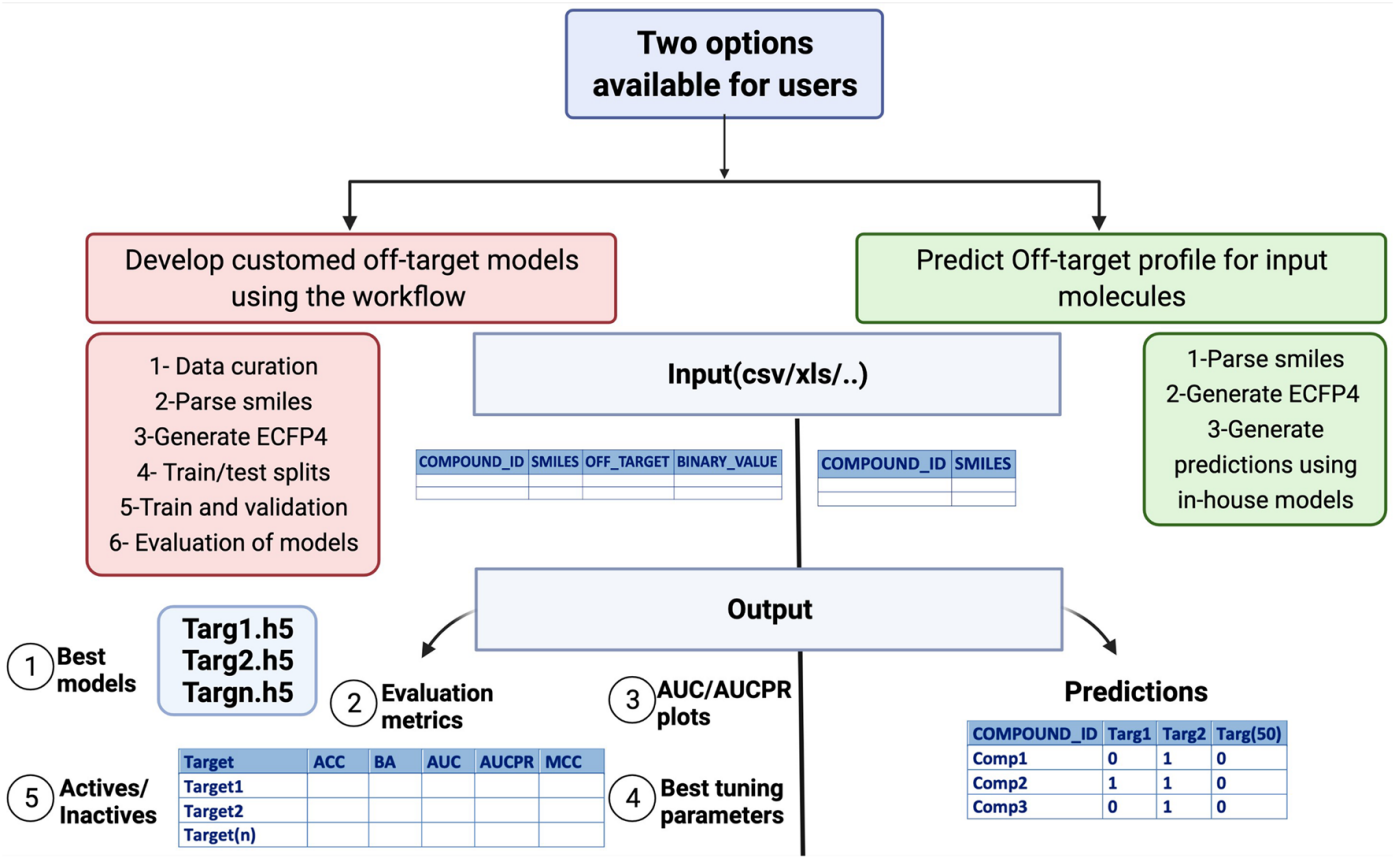
Description of the Off-targetP ML workflow

The two main purposes of the workflow are fulfilled through the two branches shown in Fig. 1:

1. Develop customized off-target models using the workflow
  This branch of the workflow trains the neural network models described in this work on any input dataset provided in a tabulated data format. The input data must contain the compound id, smiles, targets and binary activity values (active/inactive). Since duplicated smiles and measurements may be present but same compounds are usually screened against more than one target, only duplicated inter-target smiles are included and duplicated intra-target smiles are excluded. Errored or in-complete smiles are disregarded and a warning is generated. After the aforementioned data curation steps, the smiles are converted to the ECFP4 binary finger prints and used as training features for the models. Following the model training, selection of the best performing models is conducted. A complete evaluation of the models is then performed. The following output files are produced from the workflow: Best trained models in h5 format along with the corresponding tuned hyperparameters, a table with the evaluation model metrics (metrics are explained in the “Evaluation of models and overcoming bias” section) and the evaluation plots (area under the receiving operator (AUC) and precision recall (PR) curve).
  Execution can be performed from the command line by the user in three steps:
2. Finger prints preparation: *Rscript fingerprints_preparation.R input.xlsx*
3. Training and model validation: *Rscript tuning_1.R* or *sbatch tuning.sh*
4. Evaluation of the models: *Rscript evaluation.R*
5. Predict the off-target profile for input molecules
  This branch of the workflow calculates the ECFP4 fingerprints for a given smiles code, disregards errored or incomplete smiles, imports the in-house neural network in this work (in h5 format and generates the off-target panel predictions.

Execution from the command line in one step: *Rscript Off-targetP_ML.R input.xlsx.*

The workflow and the models are freely available at https://github.com/PharminfoVienna/Off-target-P-ML. Detailed instructions on how to use the workflow and to install the prerequisite packages are also provided in the same link. Details on the off-target panel, in-house models and the workflow methodologies are described below.

### Data extraction

#### In-house datasets

The off-target compounds interaction data (in terms of percentage of inhibition- [PCT]) were extracted from our in-house databases for the 50 off-targets listed in Table 1, where the target names, gene symbols/abbreviations and families are also provided. The gene symbols were retrieved from Eurofins discovery (https://www.eurofinsdiscoveryservices.com/) and are also used in the text and figures for simplification.

**Table 1:** Roche optimized off-target panel (50 targets) arranged in a descending order according to total number of compounds screened per target

Target name	Gene symbol	Target family	Compounds screened	Positives	Hit percent
Adenosine A3	ADORA3	GPCR	2834	832	29.35
Dopamine D2 short	DRD2	GPCR	2606	346	13.27
Mu-type opioid	OPRM1	GPCR	2573	364	14.14
Muscarinic M1	CHRM1	GPCR	2533	916	36.16
Seritonin 5-HT2A	HTR2A	GPCR	2391	526	21.99
Adenosine A1	ADORA1	GPCRS	2378	102	4.28
NE transporter	SLC6A2	Transporters	2360	393	16.65
Seritonin 5-HT2B	5-HT2B	GPCR	2316	823	35.53
Dopamine D1	DRD1	GPCR	2301	241	10.47
Seritonin -5HT1A	HTR1A	GPCR	2290	290	12.66
Histamine H1	HRH1	GPCR	2286	214	9.36
Muscarinic M2	CHRM2	GPCR	2253	513	22.76
Adrenergic β1	ADRB1	GPCR	2252	60	2.66
Acetylcholineesterase	ACHE	Other-enzymes	2245	418	18.61
5HT transporter	SLC6A4	Transporters	2241	799	35.65
GABA A (Cl − channel receptor)	GABRA1(CL-)	Ion-channel	2238	448	20.01
Adrenergic α1A	ADRA1A	GPCR	2210	380	17.19
Monoamine oxidase	MAOA	Other-enzymes	2204	20	0.90
Seritonin 5-HT3	5-HT3	Ion-channel	2130	50	2.34
HIV-1 Protease	HIV1-PR*	Other-enzymes	2110	46	2.18
Adrenergic α2A	ADRA2A	GPCR	2020	196	9.70
Adrenergic β2	ADRB2	GPCR	1973	76	3.85
PPARgamma	PPARG	Nuclear receptor	1955	194	9.92
Ca2 + channel (Diltiazem site)	CACNA1C	Ion-channel	1942	473	24.35
Nicotinic muscle-type	CHRNA1	Ion-channel	1899	173	9.11
Prostaglandin F	PTGFR	GPCR	1897	73	3.84
Histamine H3	HRH3	GPCR	1864	215	11.53
Xanthine oxidase	XDH*	Other-enzymes	1831	49	2.67
Glucocorticoid	NR3C1	Nuclear-receptor	1816	76	4.18
Cyclooxygenase 2	PTGS2	Other-enzymes	1799	192	10.67
Choleystokinin 1	CCKAR	GPCR	1792	139	7.75
Matrix metallopeptidase 9	MMP9	Other-enzymes	1787	38	2.12
Angiotensin receptor II	AGTR1	GPCR	1762	45	2.55
GABA-A (Benzodiazepene site)	GABRA1(Benzo)	Ion-channel	1645	329	20
Histamine H2	HRH2	GPCR	1644	123	7.48
Cannabinoid CB1	CNR1	GPCR	1609	67	4.16
Phosphodiesterase 3B	PDE3B	Other-enzymes	1563	66	4.22
ZAP70 Kinase	ZAP70	Kinases	1484	25	1.68
CDK2 Kinase	CDK2	Kinases	1465	61	4.16
GSK3 beta	GSK3B	Kinases	1394	65	4.66
Estrogen alpha	ESR1	Nuclear-receptor	1389	7	0.50
ABL1 Kinase	ABL1	Kinases	1328	236	17.77
GSK3 alpha	GSK3A	Kinases	1285	97	7.54
Androgen	AR	Nuclear-receptor	1234	86	6.96
Glutamate (PCP)	PCP*	Ion-channel	1187	6	0.50
Glycine receptor (Strychnine insensitive)	Glycine*	Ion-channel	1145	2	0.17
Nicotinic neuronal-type (alpha-BGTX insens.)	CHRNA4	Ion-channel	951	9	0.94
Angiotensin converting enzyme	ACE2	Other-enzymes	832	58	6.97
Kappa-type opioid	OPRK1	GPCR	428	89	20.79
Phosphodiesterase 4D2	PDE4D2	Other-enzymes	133	15	11.27

^*^Abbreviations for the targets and not gene symbols

The following filtration criteria was applied to the data: (a) Percentage of inhibition was measured at 10 µm concentration (b) Assay types were either radio ligand binding displacement or enzymatic assays (c) Only human recombinant receptors were used in these assays (except for GABARA1 (CL-) and (Benzo), PCP, Glycine and CACNA1C, where rat brain was used. (d) Duplicated measurements were disregarded. These safety in-vitro assays were conducted over a period of ~ 15 years (time interval for the assays collected was between September 2004 and January 2020). The panel comprised diverse target classes: 22 GPCRS, 8 Ion-channels, 5 Kinases, 4 Nuclear-receptors, two Transporters and 9 other-enzymes.

### Hit rate calculation and binary classification

A cut-off of 50% inhibition at 10 µm was drawn in order to classify the compounds’ inhibition percentages of the targets into active (≥ 50% binding) or inactive (< 50% binding) upon each target. The hit rate was then calculated according to Eq. 1 and a cut-off of 20% was specified, where a hit rate > 20% was defined as high and hit rate < 20% as low.

1 $$\frac{No. \,of \,active \,compounds}{Total \,screened}\times 100$$

### Compounds’ descriptors

Structural features of the compounds were used as descriptors for the models. Extended Circular Finger Prints with radius 4 (ECFP4) [16] were selected in this study since they are considered amongst the best performing features in bioactivity predictions [12, 17–19]. Standardization of canonical smiles and duplicates removal was performed in Pipeline Pilot (version 9.1.0) [20]. Canonical smiles were parsed into the ‘mol’ format and the 1024 bits of the ECFP4 fingerprints were generated for each compound. A matrix of 3928 compounds vs 1024 structural binary bits was obtained.

Several molecular descriptors such as Molecular weight, LogP, number of hydrogen donors, number of hydrogen acceptors, number of rotatable bonds, molar refractivity and polarizability, in addition to the number of Lipinski failures were calculated to gain an overview on the dataset. These descriptors could be used for an applicability domain implementation using a range based method (the bounding box method), where the applicability domain is defined on the basis of the minimum and maximum ranges of the mentioned descriptors [21]. An overall statistics table is provided in the supplementary material (Additional file 1: Table S1A). Calculations were done using the rcdk package in R (version 3.5.1) [22].

### Train-Test splits

Due to the high imbalance observed in the datasets, and to ensure uniform presence of positive samples in both training and test sets, a stratified activity splitting approach was used, using the caret package in R (version 3.5.1). A ratio of 80:20 was used for the training and test splits, respectively. The 80 percent training set was automatically split by Keras into 60% training and 20% validation (i.e. 60% training, 20% validation and 20% test set). Test sets were used as external held out sets and were completely excluded from the training procedure. To allow a comparison between the modelling approaches, the same training and test sets were used for each approach.

### Machine learning approaches

Two manual approaches: (Deep learning with feedforward Neural Networks and Random Forests) and three automated machine learning approaches (H2O driverless AI [23], AutoGluon Tabular [24]) and Auto-Sklearn [25]) were used to construct the models. Subsequently, a comparative analysis was performed between the five methods used, with respect to their robustness, resources consumed, and expertise needed to build the models.

Since compounds are usually screened against the suspected off-targets (not necessarily the full panel), the compounds’ off-target interaction matrix was not fully populated and comprised missing values. Thus, a single task modelling approach was adopted, where one model was constructed for each target separately.

#### Deep learning with feed forward Neural Networks

A feed forward neural network was employed using Tensorflow [26] (version 2.3) and Keras [27] packages (version 2.3) in R studio (version 1.1.456) using R (version 3.5.1). As explained above, the ECFP4 fingerprints were used as an input layer to the network to predict the binary activity of the molecules in the output layer (inactive = 0, active = 1). A sigmoid activation function was applied to the output layer while the rectified linear unit (ReLU) function was used to activate the input and hidden layers. The default threshold of the sigmoid activation function for classification was used (equals to 0.5). A drop out was applied to the input layer and the hidden layers to avoid overfitting, and a penalty was also applied to the input and hidden layer by using a kernel regularizer. The number of hidden units and dropout rate varied according to Table 2. The list of fixed parameters used in the network is presented in Table 3.

**Table 2:** Values of the grid search parameters

Parameter	Value
Hidden units	[256,512,1024,2048]
Dropout input	[0,0.1,0.2]
Dropout hidden	[0.2,0.3,0.4]
Learning rate	[0.01,0.001,0.0001]
Batch size	[64,128,256]

**Table 3:** Fixed parameters of the neural network used in the neural network

Parameter	Value
Optimizer	Adam
Loss	Binary cross entropy
Hidden layers	2
Activation	ReLu
Kernel regularizer l2	0.001
Activation output	Sigmoid

In addition to the binary accuracy function provided by Keras, a custom metric function (balanced accuracy) was created to monitor the training statistics to avoid any misleading outcomes that could be caused by the unbalanced nature of the data. Adam optimizer was employed to minimize the selected loss function (binary cross entropy), with a varying learning rate. The learning rate was further reduced when no improvement was shown in the validation balanced accuracy over 10 epochs, in order to help to reach a global minimum and to minimize random noise.

Since fitting the same neural network for 47 datasets of varying size and hit percent is a complex task, the grid search was performed on a selection of hyper parameters and a network architecture that could fit with these diverse datasets and other potential input datasets to the workflow. The variable flags used are indicated in Table 2 and were searched using the tuning function from the tfruns library provided by Tensorflow (version 2.3).

A 50% randomized sample of the grid search combinations were trained for 250 epochs (full Cartesian was not performed due to time and resource limits). However, to overcome overfitting, early stopping was applied and training was stopped when no improvement in the validation balanced accuracy was observed for 20 epochs.

In addition to the previous callbacks used during fitting the model (early stopping, reduce learning rate on plateau), model checkpoint call back was also implemented. This means that all the models were saved after each epoch, and only the model with the best validation balanced accuracy was retained. In order to ensure model generalization, a 20% random validation split was applied during the training (therefore the 80% training sets were further divided into 60% training and 20% validation).

Finally, for each target, three models were preserved, with best evaluation loss, evaluation balanced accuracy, and evaluation binary accuracy.

#### Random Forest

Random Forest models were implemented using the caret package (version 6.0–80) in R studio (version 1.1.456) using R version (3.5.1). Random Forest ensembles the outcome of multiple trees and uses bootstrap aggregation (or what is known as bagging) and randomization of predictors to improve the overall models’ accuracy. However, since Random Forest is constructed to minimize the overall error, this might result in poor prediction of minority classes. Similar to the Neural Networks, Random Forest requires optimization of several parameters, such as the number of trees and the number of variable randomly sampled as candidates at each split (mtry) and other parameters. The mtry was tuned in order to obtain the best balanced accuracy values, the number of trees were fixed to 100 and a tenfold cross validation was implemented. The algorithm was trained and tested on the same datasets as the previous methods and the models were set to be reproducible. More information on the Random Forest algorithms and implementation can be found here [28].

#### AutoML with H2O Driverless Artificial Intelligence (DAI)

H2O AutoML models were implemented using the R client driverless artificial intelligence (DAI) library (version 1.8.5.1) in R studio (version 1.1.456) using R (version 3.5.1). A license is required for the access of H2O driverless AI.

Datasets in AutoML models require less complex handling than Tensorflow models. As mentioned previously, the same data sets for each target were used, except for the difference in the shape of the dataset as explained in the supplementary information, Additional file 1. The training and test set were for each target as follows:

*Train <—dai.upload_dataset (“filepath/MuscarinicM2_train.csv”)*

*Test <—dai.upload_dataset (“filepath/MuscarinicM2_test.csv”)*

No feature engineering or hyper parameter optimization is required in H20 AutoML. Only few parameters require configuration during the training procedure, namely: accuracy, time, interpretability, and model scorers. We have chosen maximum accuracy: 10, moderate time: 6 to avoid extra-long training times and moderate interpretability: 4 to avoid extremely uninterpretable and complex models. As there is no implementation of balanced accuracy within H2O, area under the precision recall curve (AUCPR) is used for training the models, which is also considered as a suitable metric for unbalanced problems as explained in the “Evaluation metrics and overcoming assessment bias” section. Models were set to be reproducible and the default “expert settings” were used. The different algorithms implemented in H20 are: XGBoost GBM models, Light GBM models, XGBoost GLM models, TensorFlow models and RuleFit models. A final ensemble of the top performing models is automatically created in order to optimize the overall performance. More details on the models integrated in H20 and the model stacking process can be found in the H2O booklet documentation [23].

The model training is performed in as follows:

*model*<—*dai.train(training_frame* = *train, testing frame* = *test, target_col* = *“binary_activity”,is_timeseries* = *FALSE, is_classification* = *T, accuracy* = *10, time* = *6, interpretability* = *4, seed* = *30, enable_gpus* = *T, config_overrides* = *"make_mojo_scoring_pipeline* = *'off' \n min_num_rows* = *50 \n make_python_scoring_pipeline* = *'off' \n make_autoreport* = *false \n ",scorer* = *"AUCPR").*

The balanced accuracy values were calculated for the models according to Eq. 4 and were used as a final assessment metric for the models. Further details on the models evaluation procedure can be found in the supplementary information (Additional file 1).

The process was finally automated to train and retrieve the results for the models of the 47 targets.

#### AutoML with AutoGluon Tabular

Similar to the Neural Networks and H2O AutoML, we utilized open source AutoGluon (version 0.0.13) in Jupyter notebook (version 7.12.0) with python (version 3.6.5) to construct the models. AutoGluon implements several algorithms such as Neural Networks, Light Gradient boosted trees(GBM), CatBoost boosted trees, Random Forest, Extremely Randomized Trees, and k-Nearest Neighbors (kNN). A multilayer stacking strategy is adopted, where AutoGluon individually fits various models and creates an optimized model ensemble that outperforms all individual models. Further details on AutoGluons’ strategy in model training, tuning and ensembling can be found in the work of Erickson et al. [24].

Similar to H2O, AutoGluon does not require any data processing. The datasets are imported as follows:

*train_data = task.Dataset(file_path = train_filename)*

*test_data = task.Dataset(file_path = test_filename)*

A categorical selection for the training parameters is offered by AutoGluon. Allocation of the predictive accuracy, disk usage and time required for model training are defined by a list of presets. Several presets are available, such as ‘best quality’, which trains the models to obtain the best accuracy regardless of training time or disk usage, ‘best quality with high quality refit’, which is identical to ‘best quality’ but with slightly lower accuracy and higher efficiency (10 × less disk space and 10 × faster training times). All available presets are explained in details in the AutoGluon Tabular documentation [24].

We selected the option ‘best quality with high quality refit’ to ensure a reasonable balance between accuracy and efficiency. No time limit specification is needed for this option (assigned automatically by AutoGluon). Best models are also automatically stacked in this preset to produce the final model (autostack = TRUE). The parameter ‘optimize for deployment’ was also enabled in order to delete the unused/unstacked models to save disk space with no effect on the final model accuracy.

The training command lines can be summarized as follows:

*presets =  ['best_quality_with_high_quality_refit', 'optimize_for_deployment']*

*predictor* = *task.fit(train_data* = *train_data,label* = *label_column, id_columns* = * ['ID'],output_directory* = *output_directory, presets* = *presets, problem_type* = *'binary' eval_metric* = *'balanced_accuracy', verbosity* = *1,random_seed* = *42).*

AutoGluon models were evaluated on the test sets using performance metrics implemented in the sci-kit learn library (version 0.22.2). Similar to the Neural Networks and H2O, balanced accuracy values were used for the final assessment of the models.

#### Auto-Sklearn

Auto-Sklearn (version 0.14.6) was used for building the off-target models in Jupyter lab version (2.2.9) and python version(3.9.7). Similar to the previously described AutoML approaches no hyper parameter optimization was required and the model set up was simple. However, there were no predefined presets for selecting the required accuracy like Auto-Gluon and H2O, which might make it more difficult for users. On the other hand, in addition to ensemble learning, Auto-Sklearn implements a meta-learning step prior to the bayesian optimization which provides an increase in the models efficiency and thus is considered as an advantage of Auto-Sklearn [29]. Balanced accuracy was used as the model optimization metric and the time limit was set to 30 min per model and 5 min per run, with a tenfold cross validation. Evaluation of the models was done using the scikit learn package (version 0.22.2).

Command lines for data upload was similar to the Neural Networks while model training was very similar to H2O and AutoGluon and the full code can be found in the Github repository. Auto-Sklearn implements the algorithms available in the Sci-kit learn library such as AdaBoost, Gradient Boosting, kNN, Random Forests, Extremely randomized trees and others. A full documentation of the algorithms implemented, tutorials and instructions on how to use the module can be found here [25, 30].

### Evaluation of models and overcoming assessment bias

Classification models are assessed through different metrics applied to the confusion matrix of the test set, which is composed of four quadrants summarizing actual and predicted values: True positives (TP), true negatives (TN), false positives (FP) and false negatives (FN). To ensure a fair evaluation of machine learning models, the performance metrics must be carefully selected and adapted to the nature of the dataset. For example, in our study most of the targets have highly imbalanced datasets. Using the accuracy metric (calculated by Eq. 2) could be misleading, where high values could be attributed to correct prediction of the true negatives and not the true positives. Area under receiving operator curve (AUC), which measures the area under the plot of true positive rate (TPR) (calculated by Eq. 3) vs false positive rate (FPR) (calculated by Eq. 4) might also produce erroneous conclusions for the same reason.

Capturing both the true positives and true negatives is essential in off-target modeling and thus the balanced accuracy (calculated by Eq. 5), which accounts for both outcomes, was utilized as the primary metric to assess the performance of all the models and methods. Other metrics adapted to the data imbalance were also investigated through case studies, such as Mathew’s correlation coefficient (MCC) (calculated by Eq. 6), which equally weighs the four quadrants of the confusion matrix, and area under the precision-recall curve (AUCPR), which excludes the true negatives from the calculation by measuring the area under the plot of the positive predicted value (ppv) or precision (calculated by Eq. 7) vs the recall (calculated by Eq. 3). AUCPR is not affected by the true negatives and has higher sensitivity towards true positives, true negatives, and false positives. The F1 measure (Eq. 8) which is also not impacted by the true negatives count was also considered in this study.

2 $$Accuracy= \frac{\sum TN+\sum TP}{\sum P+N}$$

3 $$TPR(recall)= \frac{TP}{TP+FN}$$

4 $$FPR= \frac{FP}{FP+TN}$$

5 $$Balanced\,accuracy= \frac{\sum \frac{TP}{P}+\sum \frac{TN}{N}}{2}$$

6 $$MCC= \frac{TP\times TN-FP\times FN}{\surd (TP+FP)(TP+FN)(TN+FP)(TN+FP)}$$

7 $$Precison (ppv)= \frac{TP}{TP+FP}$$

8 $$F1= \frac{TP}{TP+\frac{1}{2}(FP+FN)}$$

### Testing our workflow and overcoming data imbalance: Six case studies

Case studies were performed to compare the prediction performance of the models constructed with in-house datasets versus new models constructed with public datasets and the impact of combining both datasets on the prediction accuracies. The six case studies were conducted for targets with a varying hit percent and dataset size: ABL1, ADRB1, AGTR1, MAO, PDE4D and PTGFR.

The off-target interaction data for these targets was extracted from the chemogenomic database “Excape” [31]. Similarly to the in-house data, only human recombinant assay target data was retrieved for the six targets. Compounds were readily classified into Actives “A” and In-actives “N”. Ambit inchikeys were provided in the Excape data and was used by the workflow for identifying the molecules.

New off-target models were developed for the six targets through pushing the Excape datasets through the left side of the Off -targetP ML workflow explained in Fig. 1 [32]. The Excape datasets were then combined with the Roche datasets of the corresponding targets and the “Combined datasets” were also pushed through the workflow. Number of compounds screened, active and in-actives are shown in Table 4. Results from the three datasets “Roche”,”Excape” and “Combined” were compared for the six target with respect to the balanced accuracies of each model.

**Table 4:** An overall comparison between the three machine learning tools used in the predictions

	Neural Networks (via Tensorflow)	Random Forest	H2O driverless AI	AutoGluon	Auto-Sklearn
Availability	Open source	Open source	License required	Open source	Open source
Time consumption (Subjective to model setting)	High	Low	High	Low	Low
Required skills	- High data science skills required in R or Python	- Basic data science skills required in R or Python	- No data science skills required for the user- interface - Minimum Python or R experience required to use the backend framework. (R/Python API client)	- Basic experience required in python	- Basic experience required in python
Supported language	R or Python	R or Python	R or Python	Python only	Python only

### Dimensionality reduction methods: Principal component analysis (PCA) and Uniform Manifold Approximation and Projection (UMAP)

A PCA was conducted to gain an overview of the chemical space covered by each dataset (Roche vs Excape) and investigate the overlap between the two datasets for the six case studies. The CDK molecular descriptors listed in Additional file 1: Table S1B were calculated using the rcdk package version (3.5.0) and rcdklibs version (2.3) in R version (3.5.1). Descriptors with zero variance were excluded. The PCA was performed using the prcomp function implemented in Stats package version (3.5.1). Details on the UMAP is presented in Additional file 3.

## Results

### Overview of the dataset activity and population

**Fig. 2:**
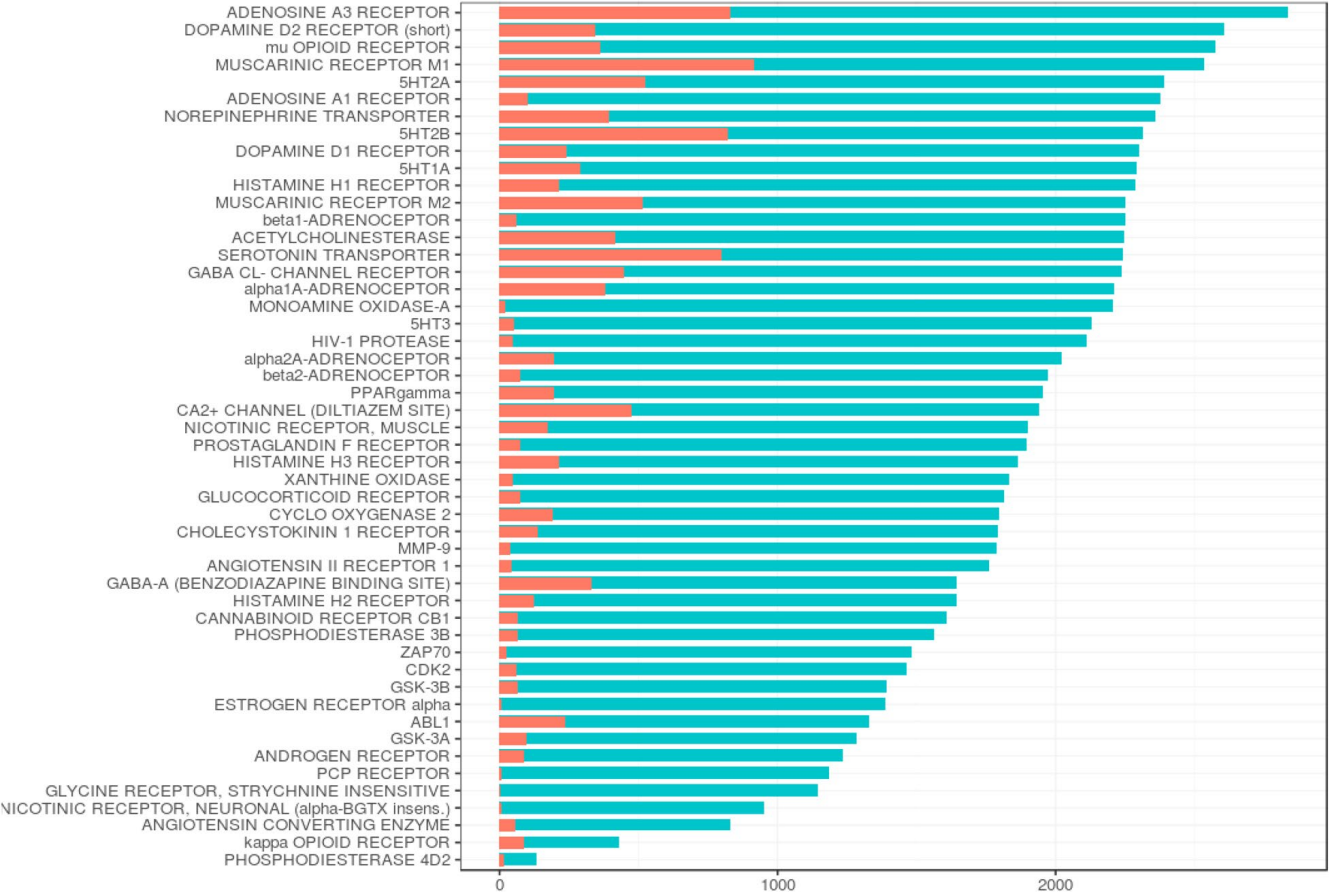
Overview of the total number of compounds screened per target and the number of active compounds within the total screened. The number of compounds is represented on the x axis and the fifty off targets are represented on the y-axis. The color code indicates the activity of compounds: pink for active compounds and blue for the total number of compounds screened

A summary of the list of targets, their symbols, total number of compounds screened (which resembles the dataset size of each target), number of active compounds, and the hit percent for each target and target families are given in Table 1. As illustrated in Fig. 2, not all compounds were screened against each target and a high variability is shown within the targets in terms of dataset size and hit percent.

Most of the datasets had a considerable number of compounds, for example, among the 50 targets, 43 targets had > 1000 compounds and only 4 targets had < 1000 compounds. Only two datasets were rather small compared to the other targets OPRK1 = 428 compounds and PDE4D = 133 compounds.

Only 10 targets showed a hit percent higher than 20 (e.g. HTR2A 35.65 CHRM1 36.16%, HTR2B 35.53%), while 40 targets showed a hit percent lower than 20%. A few amongst these 40 targets exhibited a very low hit percent (e.g. HIV1-PR 2.18%, ADRB1 2.66%, AGTR1 2.53%). Other targets like PCP, Glycine and CHRNA4 exhibited further lower hit rates (0.1–0.5%) and the corresponding models constructed exhibited extremely poor performance (Additional file 2: Table S3). They were therefore excluded from the analysis and the final dataset consisted of 3928 unique compounds and 47 targets.

The impact of both factors (dataset size and hit percent) on the model performance is discussed in the “Technical and biological factors affecting model performance” section.

### Comparison of models’ performance between Keras, H2O and AutoGluon Tabular

#### Overall performance

**Fig. 3:**
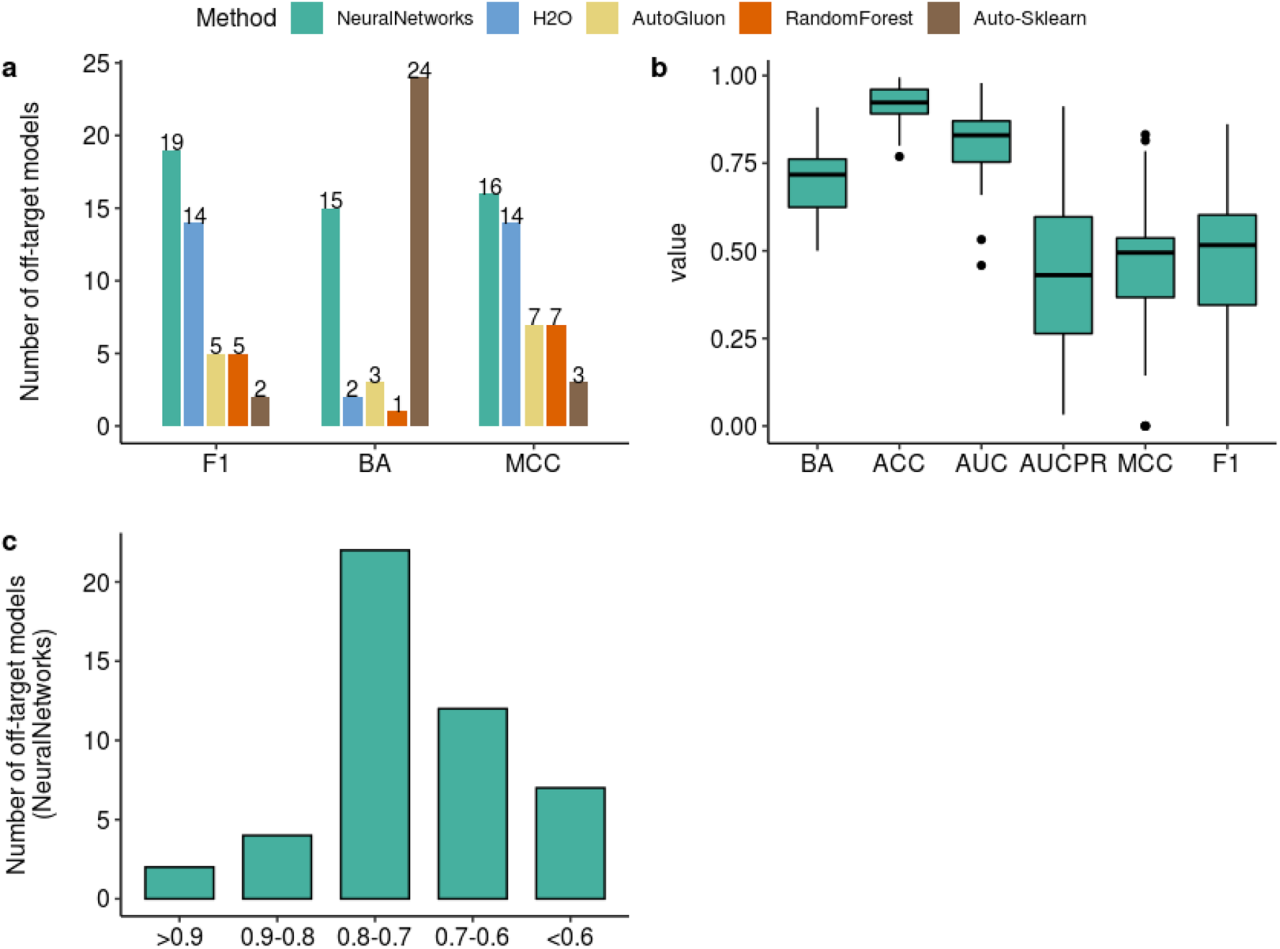
An overall comparison between the performance of the five machine learning methods used for the construction of the off-target models, an overview of all the performance metrics and the balanced accuracy ranges for the neural networks. **a** A bar plot comparing Neural Networks, H2O, AutoGluon, RandomForest and Auto-Sklearn (x-axis) with respect to the number of targets each method scored the highest Mathews Correlation Coefficient(MCC), Balanced Accuracy(BA) and F1 (y-axis).The bars are color coded according to the machine learning method and the number of the targets each method scored highest is indicated on the top of each bar. **b** A box plot comparing the values (y-axis) of the different performance metrics (x-axis) for the Neural networks method. **c** A bar plot displaying the number of off-target models (y-axis) falling under each balanced accuracy range (x-axis) for the neural networks method

An overall view of the highest scoring machine learning method for each target model in terms of balanced accuracy, MCC and F1 is represented in Fig. 3a. Neural Networks scored a higher F1 and MCC than the rest of the methods for 19 and 16 targets respectively. H2O ranked second where it scored highest for 14 targets with respect to both MCC and F1. AutoGluon and Random Forest showed similar performance while Auto-Sklearn ranked lowest with respect to these two important metrics. While 24 Auto-Sklearn models exhibited the highest balanced accuracy amongst the rest of the methods, it does not qualify for an overall best performance due to the poor performance with respect to the MCC and F1. In addition to the outperformance of the Neural Networks in the F1 and MCC measures, it outperformed the remaining methods in the balanced accuracy as well (scoring the highest for 15 targets). Therefore the Neural Networks seem to have a better overall performance and can be considered as the top performing method in the context of this study.

Performance of the Neural Networks with respect to different evaluation metrics was inspected and discrepancies were observed as seen in Fig. 3b. Accuracy followed by AUC showed optimistic values with mean values 0.91 and 0.81 respectively. As previously described in the methods section, these metrics can be biased for highly imbalanced datasets. On the other hand, mean values for F1, MCC and AUCPR were very close (0.48,0.44,0.45 respectively) and were less optimistic than the Accuracy and AUC values. The mean value for the balanced accuracy was lower than Accuracy and AUC but higher than the MCC and F1 (mean BA = 0.7).

In the next section we mostly use the balanced accuracy to explore the individual discrepancies in target models with respect to each method. However it should not always be exclusively utilized in models’ assessment and is preferably associated with other metrics. A detailed analysis of all the performance metrics (Balanced Accuracy, Accuracy, AUC, AUCPR, MCC and F1) is therefore provided in the supplementary information for the Neural Networks, H2O, AutoGluon, Random Forest and Auto-Sklearn (Additional file 2: Table S3A–E respectively).

In Fig. 3c the balanced accuracy range of the targets is illustrated for the highest scoring method, Neural Networks. The majority of the neural network models were successful, where the balanced accuracy was between 0.7 and 0.8 for 22 of the targets, between 0.8 and 0.9 for 4 targets and exceptionally high (> 0.9) for 2 targets (in total 28 targets with balanced accuracy more than 0.7). Few targets had a moderate balanced accuracy between 0.6 and 0.7 (12 targets) and 7 targets were not well predicted and exhibited a low balanced accuracy (less than 0.6).

**Fig. 4:**
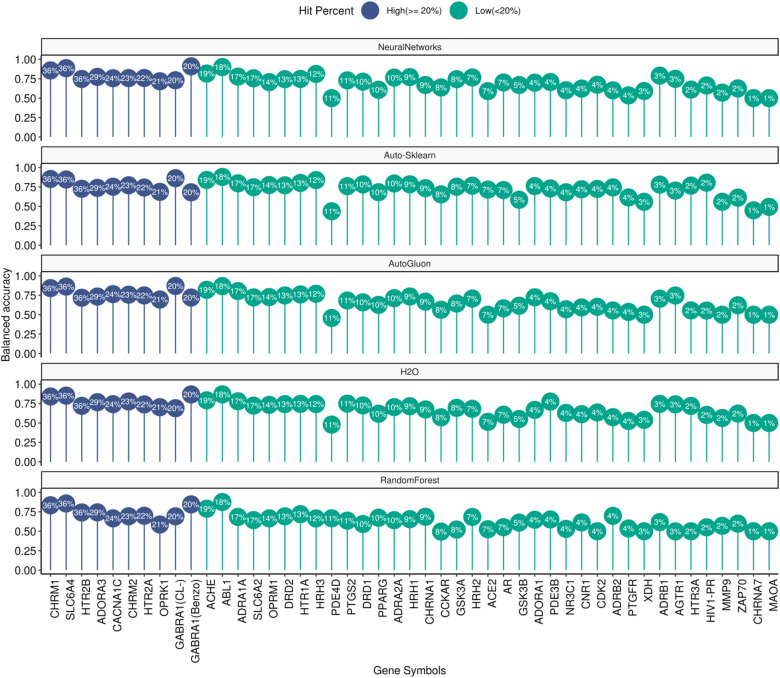
Lollipop chart representing the balanced accuracy of each target model for each method. Each subplot represents one of the methods used to build the models (Neural networks, Auto-Sklearn, AutoGluon, H2O and Random Forest). The gene names/abbreviations of each target model are represented on the x-axis and the corresponding balanced accuracy values on the y axis. Lines are color coded according to the targets’ hit percent category and the numerical values of the hit percent are indicated inside the circles

The variability within the models’ performance is investigated through a detailed insight into the balanced accuracy values obtained for each target (via the five methods) along with the factors that might have affected the performance, such as the hit percent. The values of the balanced accuracy for each target model and the respective hit percent values are displayed in Fig. 4 (Balanced accuracy values for the five methods is present in the supplementary information (Additional file 2: Table S3). Most of the high hit percent targets were predicted with a balanced accuracy higher than 0.7 for all methods except for the OPRK1 (AutoGluon = 0.69, Auto-Sklearn = 0.68 and Random Forest = 0.58). Random Forest showed balanced accuracy less than 0.7 for other high hit percent target (e.g. CACNA1C = 0.66). Differences between the five methods were seen in the performance of the GABRA1 (CL-) channel models, where AutoGluon and AutoSklearn (both BA = 0.86) outperformed Neural Networks, H2O, and Random Forest ( 0.73, 0.690 and 0.692 respectively).

Despite the similar hit percent values between the three targets: PDE4D, PTGS2 and HRH3 (11.2, 10.6 and 11.5 respectively), All methods (except Random Forest) showed a poor performance for the PDE4D target (mean value BA = 0.46) while succeeded in PTGS2 (Auto-sklearn,H2O and Neural Networks = 0.76, 0.74 and 0.72 respectively) and HRH3 (Auto-Sklearn = 0.83 and Neural Networks = 0.81). This could be attributed to the small sample size of the PDE4D used to construct the model (n = 133) versus those for PTGS2 (n = 1799) and HRH3 (n = 1864). Interestingly the Random Forest model showed the highest balanced accuracy for PDE4D (BA = 0.66). Other targets like OPRK1 exhibited a small dataset size (n = 423), however, the relatively high hit percent of OPRK1 (20.79%) provided a model with at least moderate balanced accuracy (an average of 0.69 across all methods excluding the Random Forest method where BA = 0.58).

18 targets with low hit percent (< 20%) were predicted with a relatively good balanced accuracy (> 0.7) for Neural Networks. Despite a low hit percent, some targets showed very high balanced accuracies for all the methods (e.g. ABL1, hit rate = 17.7% and mean BA = 0.87). Two targets had a significantly lower hit percent than the others: Angiotensin receptor 1 (AGTR1) and beta-1 adreno receptor (ADRB1) (hit percent = 2.5 and 2.6 respectively). Both targets showed a surprisingly good balanced accuracy (mean balanced accuracy of four methods excluding Random Forest ~ 0.74 for both targets). Both targets are class A GPCR receptors and have a relatively large dataset size (ADRB1, n = 2252 and AT1, n = 1762). On the other hand, the PTGFR, (which is also a class A GPCR receptor) had a low hit percent but in contrast to AGTR1 and ADRB1, no high balanced accuracy was observed (mean balanced accuracy = 0.54). The PTGFR receptor is involved in allosteric binding which in addition to the low hit percent, might be another reason for the low model predictivity.

The same analysis was performed with respect to the F1 measure and is provided in the supplementary material (Additional file 3: Fig S1), where it uncovered the poor performance for some of the methods specifically with respect to the targets with low hit rates.

### Summary on the technical and biological factors impacting prediction performance

#### Technical factors (hit rate and data set size)

**Fig. 5:**
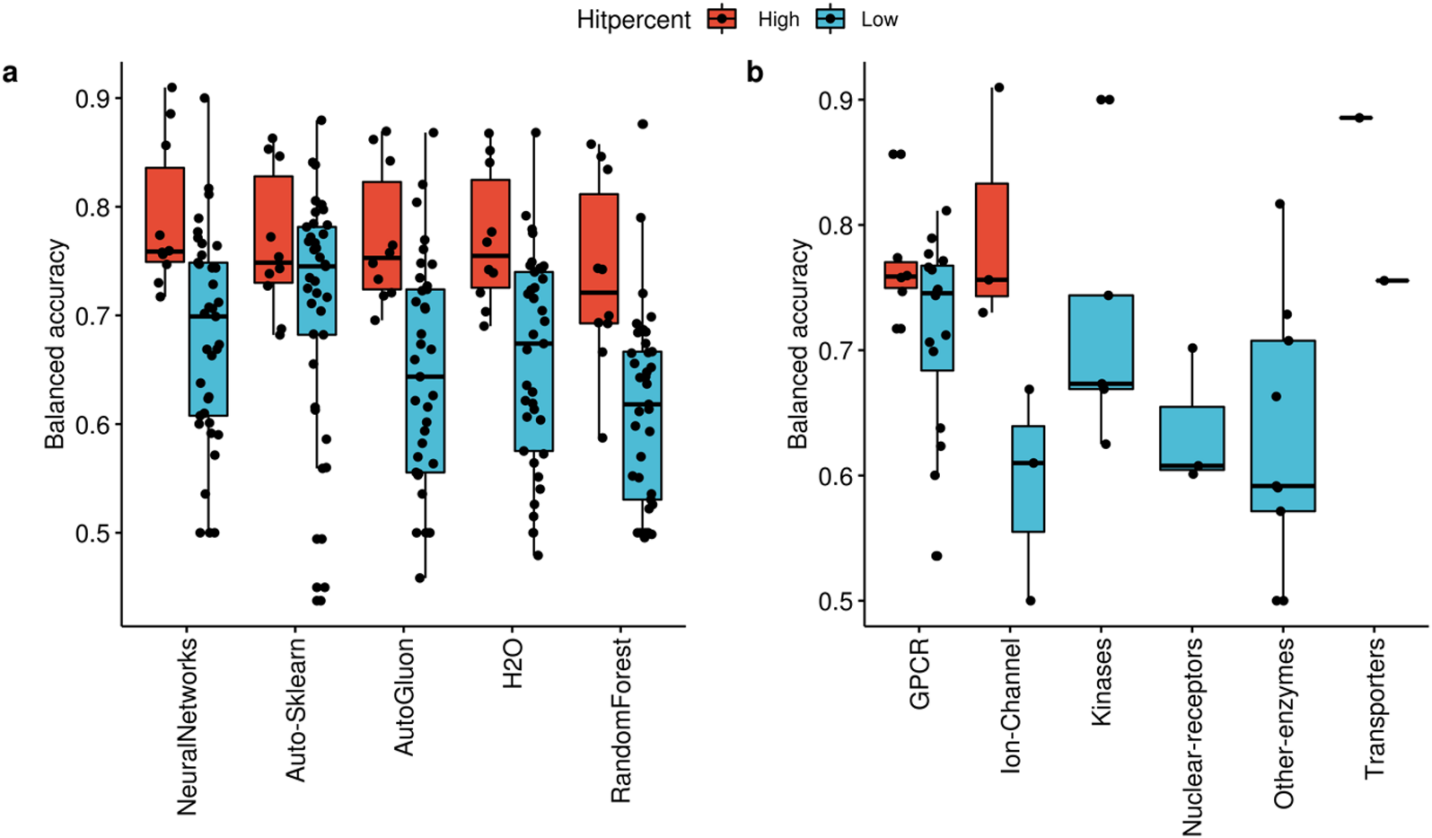
Box plots comparing the overall balanced accuracy for high and low hit percent target groups, with respect to t the **a** Machine learning methods and **b** Target protein classes. The five methods are represented on the x axis and the balanced accuracy values on the y axis. The red box plots represent the high hit percent target groups and the blue box plots represent the low hit percent target groups. The High hit percent target groups achieve higher balanced accuracies irrespective of the method used. The target classes are represented on the x axis and the balanced accuracy values of the neural networks on the y axis. For some target classes, no significant difference is seen in the overall balanced accuracy between the high hit percent and low hit percent groups (e.g. GPCR class) while for other classes (e.g. Ion-channels), a significant difference is seen in the overall balanced accuracy between the high and low hit percent groups. The hit percent is represented on the x axis and the balanced accuracy on the y axis. Each circle represents a target. The circles are color coded according to the target classes. The size of the circles varies according to the dataset size

Figure 5a shows the effect of the hit rate on the model performance in terms of balanced accuracy and the overall differences in the five methods performance with respect to the two groups (high and low hit rate groups). The same figure is displayed with respect to the F1 measure in the supplementary material (Additional file 3: Fig S2). A statistically significant difference is shown between the high and low hit rate groups with respect to the Balanced accuracy for all the methods except AutoSklearn (Fig. 5a) (Wilcoxon rank test p-value = 0.001, 0.004, 0.002, 0.0005 and 0.286 for AutoGluon, H2O, Neural Networks, Random Forest and Auto-Sklearn respectively). Auto-Sklearn showed similar mean balanced accuracy values for the high and low hit rate groups (0.76 vs 0.71). All the methods performed similarly in predicting high hit rate targets. Neural Networks showed the best performance for the high rate groups (mean balanced accuracy = 0.79) followed by an equal performance of H20 and AutoGluon (mean balanced accuracy = 0.77 for both methods) and finally RandomForest (Mean BA = 0.73). Regarding the low hit rate category, the mean balanced accuracy dropped by more than 10% for the four methods. The same trend is observed where Neural Networks again showed a slightly better performance than the other methods in predicting the low hit rate targets, followed by H2O, AutoGluon and finally Random Forest(mean balanced accuracy of 0.68, 0.66, 0.64 and 0.61 respectively).

A statistically significance difference was seen between the high and low hit rate groups for all the methods with regards to the F1 measure and both the Neural Networks and H2O yielded the highest F1 mean values among other methods for both the high (mean F1 = 0.695, 0.683) and low hit percent groups (mean F1 = 0.42 and 0.44) (Additional file 2: Fig. S2a). These results are in agreement with the performance ranking discussed in Fig. 3 where the Neural networks were considered overall the best performing method.

#### Biological factors

The impact of the target protein families is displayed in Fig. 5b, where little or no difference is seen between the high hit percent and the low hit percent group in the GPCR class for the balanced accuracy. On the other hand, a large difference is seen between the high hit percent and low hit percent group for the Ion-channel class. The high hit percent Ion-channel CACNA1C and GABRA1 receptors (CL − and Benzo) had an average balanced accuracy of 0.808, while the low hit percent Ion-channels: Nicotinic receptors (CHRNA1, CHRNA4) and HTR3 had a much lower balanced accuracy (average = 0.57). With regards to the ‘Kinases’ class, despite that all the targets had a low hit percent, the average balanced accuracy was equal to 0.72. On the other hand, all the targets in the Nuclear receptor class also belonged to the low hit percent group but still had a low average balanced accuracy (0.63). Targets in the enzymes class also belong to the low hit percent group and showed a wide balanced accuracy distribution (from 0.50 till 0.81). The transporter class comprised two targets, with a high balanced accuracy for the high hit percent target (SLC6A4, hit percent = 35.65%, BA = 0.88) and the low hit percent target (SLC6A2, hit percent = 16.65%, BA = 0.76). Differences were seen between the two hit rate groups for all the target families including the GPCRs with respect to the F1 measure (Additional file 2: Fig S2b).

**Fig. 6:**
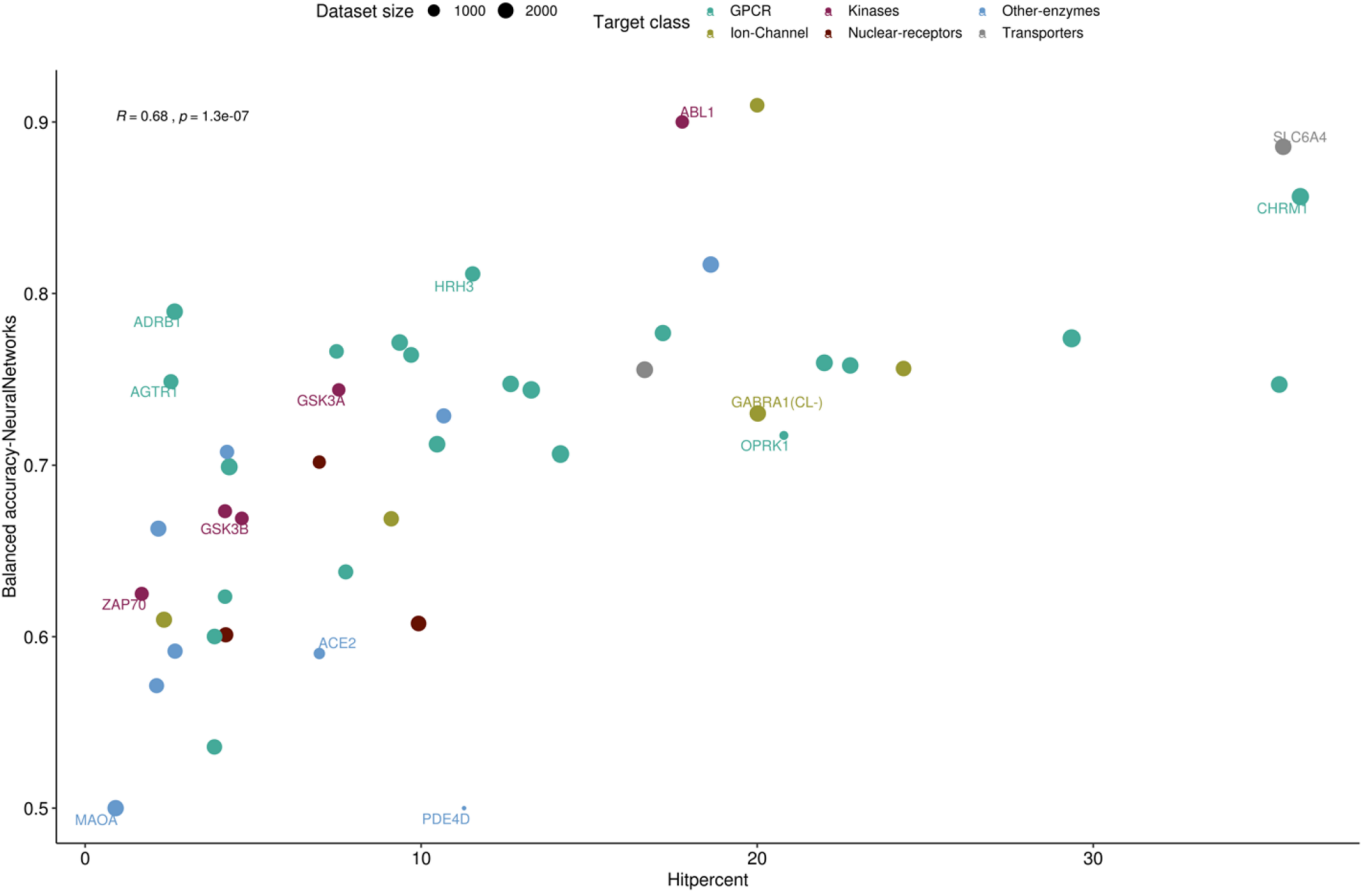
Scatterplot displaying the impact of the hit percent, data set size and target class on the balanced accuracy of the individual target models

Both, the correlation between the hit percent and the balanced accuracy of the neural network models as well as the impact of the target type on the model performance is displayed in Fig. 6. It can be deduced that some target families such as GPCRs, kinases, and transporter, might be easier to predict than e.g. the ion-channels and nuclear receptors (Fig. 6). However, further investigation into the impact of the target classes on a larger scale would be needed to validate this assumption. Other factors such as allosteric binding of targets and the presence of co-enzymes should also be explored.

There was no systematic impact of the dataset size seen on the prediction accuracy of the models, as most of the datasets comprised > 1000 compounds. However, target models with small dataset size showed either poor (PDE4D) or mediocre performance (e.g. OPRK1).

#### Implementation time and resources

The Neural Networks and Random Forest were trained using 3 GPUs and 18 CPUs on an Intel(R) Xeon(R) Gold 6148 CPU @ 2.40 GHz machine with 40 cores and 768 GB memory, AutoGluon and Auto-Sklearn were trained on an Intel(R) Xeon(R) CPU E5-2680 v3 @ 2.50 GHz machine with 24 cores and 256 GB and finally the H2O models were trained using an amazon web services (AWS) virtual machine (m5.2xlarge instance) with 8 CPUs and 32 GB memory.

For Neural Networks the calculation took between 3 and 4 h per target, resulting in a total of 188 h for the 47 targets. H2O training ranged from 1.35 h to 9.50 h depending on the target, resulting in a total training time of 173.81 h. Auto-Sklearn took 30 min per target resulting in a total of 23.5 h. Random forests consumed 7.5 h in total since we did not perform an extensive hyper parameter optimization. By far the most rapid method was Random Forest followed by AutoGluon, which consumed around 20 min per target (around 16 h in total for all the models). An overall comparison between the three methods with respect to availability, time consumption, required skills and supported languages is shown in Table 4. The time consumption described in Table 4 corresponds to the previously described training settings of each tool and is highly subject to change depending on these settings.

### Results for the six case studies

It can be deduced from the previous results that data imbalance was one of the most impactful factors on the prediction accuracies of the off-target models. Although the Excape datasets were smaller in size than the Roche ones, they were more balanced as it can be seen from the number of actives and inactives for each dataset (Table 5). Three important conclusions can be drawn from this table: Firstly, our workflow achieved successful predictions for the six public Excape datasets.

**Table 5:** Overview of the activity and balanced accuracy values of the Roche, Excape and the Roche-Excape combined datasets for the six case studies

Target	Dataset								
	Roche			Excape			Combined		
	A	N	BA	A	N	BA	A	N	BA
ABL1	236	1092	0.9	1701	141	0.684	1937	1233	0.88
ADRB1	60	2192	0.789	1161	99	0.683	1221	2289	0.92
AGTR1	45	1717	0.748	667	546	0.939	712	2259	0.93
MAOA	20	2184	0.5	683	700	0.84	703	2280	0.85
PDE4D	15	118	0.5	461	97	0.868	476	215	0.91
PTGFR	73	1824	0.53	91	14	0.75	164	1834	0.56

“A” stands for active and “N” stands for non-active compounds

Secondly, for four out of six case studies the imbalance problem was overcomed through combining both datasets. Thirdly, most of the balanced accuracies for the combined dataset were better than the individual datasets.

**Fig. 7:**
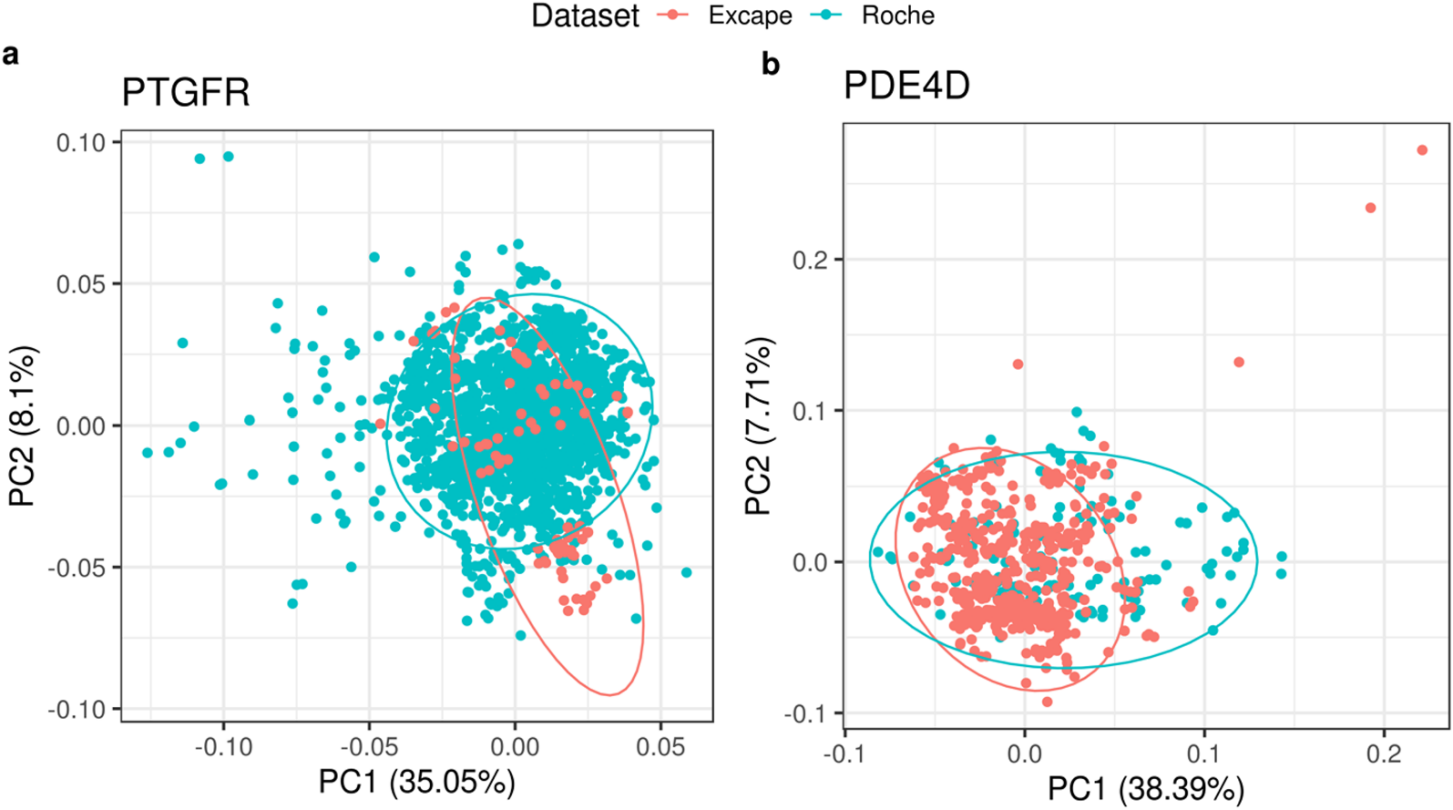
PCA of the Roche-Excape combined datasets for the two targets: **a** PTGFR and **b** PDE4D. Pink dots represent the Excape compounds, blue dots represent the Roche compounds. The x axis and y axis represent the first two components of the PCA and the percentages indicated represents the variance explained by each component. The ellipses represent the confidence intervals and is implemented through stat_ellipse function in R ggplot2 package

An exception to that was the PTGFR case study, where the imbalance ratio of active to inactive was improved from 73/1824 to 164/1834 but was not completely resolved. Despite doubling the number of actives, the combined PTGFR model did not show a better balanced accuracy than the Roche PTGFR model. Less overlap was seen in the chemical space of the Roche vs the Excape PTGFR datasets in the PCA plots (Fig. 7a) and the Umap plot (Additional file 3: Fig. S3f). Many of the Roche compounds were not covered by the chemical space of the Excape compounds, which might explain the absence of improvement in the models.

For PDE4D, a significant improvement was observed in the balanced accuracy, from 0.5 (Roche) to 0.91 (combined). In the PDE4D case study, combining the datasets resolved two issues (i) the data imbalance and the (ii) small dataset size. In addition to that, a considerable overlap is seen between the two chemical spaces in Fig. 7b.

No improvement was seen for ABL1 Roche model upon combining the dataset with Excape, although the hit percent was significantly higher in the combined dataset.

The remaining PCA plots of the targets can be seen in Additional file 3: Fig. S3 which show a good but not a full overlap between the datasets for each target. Same conclusions can also be drawn from the Umap plots of the six case studies provided in Additional file 3: Fig. S4. The full evaluation metrics table for the individual Excape and the Roche-Excape combined models is also presented in Additional file 3: Tables S4, S5 respectively.

Given the heterogenicity of the combined datasets and the difference in the assays, the question arises whether the combined models are as reliable as the individual models and the confidence we can place in the robustness of these models.

## Discussion and next steps

The goal of this work was to construct an open-source workflow that predicts the in-house off-target profile for any chemical structure and also build custom models for any given dataset with a minimal effort required from the user and with an extended implementation to public data.

To develop this workflow, we have explored and compared several techniques that can alleviate challenges such as data scarcity, imbalance, and prediction assessment bias. One of the key learnings was the importance of analyzing and understanding the nature of the dataset and the impact of applying certain techniques at different stages of modelling: (1) Implementation of stratified activity splitting of the datasets prior to model construction (2) Implementation of early stopping, model checkpoints and training on an unbiased performance metric (e.g. balanced accuracy), grid search on hyper parameters during model construction, and finally (3) Choosing the best model according to again an unbiased metric after model construction and while assessing the model performance. Deploying such techniques showed an improvement in the models and provided realistic evaluation of the model performance.

Another important consideration while handling an imbalanced dataset is the choice of the most compatible performance metric with respect to the nature of the dataset (i.e. balanced or imbalanced) and the costs of the false predictions (e.g. high costs of False positive or False negatives). While the balanced accuracy could give us an equal indication on the model performance with respect to true positives and the true negatives, the F1 measure (which excludes the true negative from the assessment) was more important for highly imbalanced datasets where it gave us an overview on the precision and recall measures of the models and uncovered poor performance of models with a high true negative rate.

We have also explored different machine learning approaches in this work, such as manual construction of the models using Neural Networks and automated model building through three automated machine learning tools, AutoGluon, H2O and Auto-Sklearn. Although Neural Networks was the best performing method for the majority of the target models, setting up a neural network framework required several tactics and expertise to manually implement hyper parameter optimization in order to create robust models. Once the grid search parameters are chosen, the network has to exhaust the possible combinations, which is extremely time consuming. Consequently, we were not able to tune all the neural network parameters, such as the sigmoid function prediction threshold, which might have been helpful to overcome the data imbalance, but would have hugely expanded the calculation time and memory. On the contrary to Neural Networks, AutoML did not require any hyper parameter tuning and was less time consuming, since time allocation was one of the configuration settings that a user can choose.

Some differences were observed between the three AutoML tools, such as the configuration parameters. For H2O, one has to manually set a numerical value (ranging from 0 to 1) for the accuracy, time and interpretability of the model training. This gives quite a large flexibility in the model construction but it might be more difficult to tweak and assess the best settings needed for each dataset. The configuration settings of AutoGluon were categorical, which could be simpler to choose, either high quality models with limited time allocation or medium quality models with less accuracy but very rapid implementation. As for Auto-Sklearn, there were no categorical or discrete values to set the models’ required accuracy, however, attaining the best accuracy was dependent on the maximum time allowed for running the models. It is recommended by Auto-Sklearn to set the time allowance for 1 day per model which is highly time consuming. On the other hand, H2O offers a user-friendly interface in addition to a package implementation in R or Python, while AutoGluon and Auto-Sklearn can only be implemented in Python. The three tools can be configured for running preliminary model training with minimal time and resources consumption.

Benchmarking of AutoML approaches has been previously performed on various datasets to assess their performance on classification versus regression tasks [33].However this benchmarking did not cover datasets similar to those adopted in this work (e.g. compound-target interaction datasets) and a lack is generally observed with respect to AutoML benchmarking in the field of cheminformatics. In this work, AutoML methods performed fairly well with respect to our binary classification task, however, more work is needed to explore their performance with respect to regression problems in this field.

On a general note, AutoML can also be used as a quick and practical guide for other tailored machine learning approaches, for example HTR3 was more accurately predicted with H2O AutoML than with Neural Networks, indicating that this is not a dataset issue and that the Neural Networks were simply not able to converge for this specific target and could be further improved.

However, regardless how simple or exhaustive the machine learning method is, the prediction failed in some cases and this was due to the nature of the dataset. If there is extreme imbalance in the dataset, hyperparameter optimization will likely not improve the prediction.

Therefore we have investigated the combination of our in-house data with public data (Excape) to overcome this issue and to evaluated this approach on six case studies. Although the models were improved for four out of six targets and the chemical spaces of the models were expanded, differences in assay measurements could be a potential cause for noisy predictions and decreased reliability of the models. The models’ robustness was seen as a more important criteria than the chemical space expansion. Nevertheless, the moderate to high balanced accuracy values (0.68–0.92) of the individual Excape models confirms the overall success of our workflow in off-target predictions of public datasets.

Data augmentation techniques such as oversampling, through either generating multiple conformations of the scarce active molecules (COVER) [34] or creation of artificial samples (SMOTE) [35] could also help overcome the data imbalance. Another possibility would be perceiving the data imbalance from a different aspect. That is to say, we can consider the active molecules as dataset outliers, which the algorithm is trained to detect. This technique is called anomaly detection [36, 37], which have been widely and successfully used in addressing real world data problems, medical imaging and clinical research [38–41]. However, implementing these methods would have been far beyond the scope of this manuscript.

Finally, since discrepancies in the prediction success were observed for different gene families (for example the GPCR family models performed well regardless of the hit percent, while in the ion-channel family models were highly dependent on the hit percent), incorporating protein descriptors into the models could be of great value. A chemogenomic neural network was described in the work of Playe et al., where the authors achieved successful bioactivity predictions through combining both protein and molecule encoders in a feed forward neural network [42]. Methods based on this approach are described as Proteochemometric modelling (PCM) [43]. Other studies have proposed pharmacological space augmentation via PCM and autoencoder models [44].

In our case, not only can we encode the protein descriptors and feed it into the network, but it could also be of great value to exploit target models with high prediction accuracies and extrapolate these models to the low performing targets included within the same protein family, possessing similar pocket sequence or similar inhibition mechanisms. This approach could be perceived as transfer learning, where a model is pretrained on a first task (balanced target dataset in our case) and then repurposed it for another related task (another imbalanced target dataset). Previous studies have shown the success of transfer learning success in bioactivity predictions [45, 46]. Multi-task learning, introduced by Rich Caruana in 1997 [47] is another valuable ML approach that has improved the predictive performance of compound-target activity models [48, 49]. In the context of this work, a multi-task deep learning network can be used where several properties of the compounds are co-modelled and used as predictors for the binary activity values. Another approach is modelling related protein-targets simultaneously where the network information is shared between these targets leveraging the missing data from one target to the other. Data imputation could also be used to overcome the missing activity values, however the high percentage of missing values in our off-target compound interaction matrix could lead to inaccurate imputations [50].

For both multi-task and transfer learning, task relatedness should be carefully assessed and might be one of the challenges faced in this field. In addition to that, both methods require coding expertise to be implemented and are not offered within the current AutoML tools [51].

Overcoming the challenge of data scarcity and imbalance through attempting some of the previously mentioned approaches, transfer learning in particular, represents the next steps in our off-target prediction framework.

## Conclusion

In this work we developed an open source workflow for off-target predictions based on Roche in-house data. The user can choose to generate predictions for any given chemical structure using the in-house models or to build custom off-target models using the workflow. The constructed off-target models implemented in our workflow can be deployed in the drug discovery pipeline and guide chemists throughout compounds’ design prior to synthesis, with relatively good prediction accuracies achieved for the majority of the in-house panel. Reasons behind the poor performance of some of the target models were identified and techniques to overcome these issues were proposed. Enriching the in-house models with public data alleviated the imbalance issues, however confidence in these models remains uncertain. Successful predictions were also achieved by the Off-targetP ML workflow for public datasets. Finally, the overall comparison between the machine learning approaches explored in this work showed that Neural Networks outperformed the three AutoML methods(AutoGluon, H2O and Auto-Sklearn), which, however achieved similar performance for many of the targets and therefore might provide a quick, practical, and user-friendly alternative to manual model building in the future.

## Supplementary Information

**Additional file 1: Table S1.A** Ranges of the molecular descriptors used for the applicability domain. **Table S1.B** List of CDK descriptors used for the PCA and UMAP. Supplementary paragraph on training steps for H2O and AutoGluon Tabular. **Table S2.** Neural networks performance metrics for the three target excluded from the analysis due to their poor performance.

**Additional file 2: Table S3.A–E** Comparison of different performance metrics for the Neural Networks, H2O, AutoGluon, RandomForest and AutoSklearn methods respectively.

**Additional file 3: Fig. S1.** Lollipop chart representing the F1 values of each target model for each method. **Fig. S2.** Box plots comparing the overall F1 values for the high and low hit percent target groups, with respect to (a) machine learning methods and (b) Target protein classes. **Fig. S3.** PCA of the Roche-Excape combined datasets for thefour targets (a) ABL1 (b) ADRB1 (c) AGTR1 (d) MAOA. Supplementary paragraph on Uniform Manifold Approximation and Projection (UMAP). **Fig. S4.** Umap of the Roche-Excape combined datasets for the six case studies. Table S4. Comparison of different performance metrics for the Excape models. **Table S5.** Comparison of different performance metrics for the combined Roche-Excape models.

## Data Availability

The Off target-ML workflow is freely available for use and download at the Github repository: https://github.com/PharminfoVienna/Off-target-P-ML. The workflow is demonstrated on the public Excape datasets which can be directly downloaded from the following link: https://solr.ideaconsult.net/search/excape/ (compiled Excape datasets and demonstration results are also deposited in the Github folder). Detailed instructions on how to use the workflow and download the required packages is provided. The code required for the AutoML(data preparation and model construction) and Random Forest are also available in the repository. The deep learning models implemented in the workflow can be directly downloaded in the h5 format from the repository.

## Declarations

### Disclaimer

This paper reflects only the authors´ view. Innovative Medicines Initiative 2 Joint Undertaking is not responsible for any use that may be made of the information it contains.
