# Supplementary material for: Off-targetP ML: an open source machine learning framework for off-target panel safety assessment of small molecules
Source: J Cheminform. 2022 May 7;14:27. doi: 10.1186/s13321-022-00603-w (PMC9077900; doi:10.1186/s13321-022-00603-w)
Supplement: Supplementary file 1 — Additional file 1: Table S1.A Ranges of the molecular descriptors used for the applicability domain. Table S1.B List of CDK descriptors used for the PCA and UMAP. Supplementary paragraph on training steps for H2O and AutoGluon Tabular. Table S2. Neural networks performance metrics for the three target excluded from the analysis due to their poor performance. [file 13321_2022_603_MOESM1_ESM.docx]

**Table S1.A** Ranges of the molecular descriptors used for the applicability domain.

| **Descriptor** | **min_desc** | **max_desc** | **mean_desc** |
| --- | --- | --- | --- |
| MW | 76.054 | 7618.383 | 474.105 |
| LipinskiFailures | 0 | 5 | 0.638 |
| nRotB | 0 | 178 | 6 |
| nHBDon | 0 | 54 | 2 |
| nHBAcc | 0 | 200 | 7 |
| ALogP | -25.0343 | 10.9456 | 3.022 |
| ALogp2 | 1.59998E-07 | 626.716 | 12.704 |
| AMR | 14.9072 | 1685.555 | 121.859 |
| apol | 8.231 | 993.410 | 68.177 |

**Table S1.B** List of CDK descriptors used for the PCA and UMAP

| **CDK descriptors** |
| --- |
| FractionalCSP3Descriptor |
| SmallRingDescriptor |
| FractionalPSADescriptor |
| ZagrebIndexDescriptor |
| XLogPDescriptor |
| WienerNumbersDescriptor |
| WHIMDescriptor |
| WeightDescriptor |
| VAdjMaDescriptor |
| VABCDescriptor |
| TPSADescriptor |
| RuleOfFiveDescriptor |
| RotatableBondsCountDescriptor |
| PetitjeanShapeIndexDescriptor |
| PetitjeanNumberDescriptor |
| MomentOfInertiaDescriptor |
| MDEDescriptor |
| MannholdLogPDescriptor |
| LengthOverBreadthDescriptor |
| LargestPiSystemDescriptor |
| LargestChainDescriptor |
| KierHallSmartsDescriptor |
| KappaShapeIndicesDescriptor |
| HybridizationRatioDescriptor |
| HBondDonorCountDescriptor |
| HBondAcceptorCountDescriptor |
| GravitationalIndexDescriptor |
| FragmentComplexityDescriptor |
| FMFDescriptor |
| EccentricConnectivityIndexDescriptor |
| CPSADescriptor |
| ChiPathDescriptor |
| ChiPathClusterDescriptor |
| ChiClusterDescriptor |
| ChiChainDescriptor |
| CarbonTypesDescriptor |
| BPolDescriptor |
| BasicGroupCountDescriptor |
| AutocorrelationDescriptorPolarizability |
| AutocorrelationDescriptorMass |
| AutocorrelationDescriptorCharge |
| AtomCountDescriptor |
| AromaticBondsCountDescriptor |
| AromaticAtomsCountDescriptor |
| APolDescriptor |
| ALOGPDescriptor |
| AcidicGroupCountDescriptor |

### ***AutoML with H2O Driverless Artificial Intelligence (DAI)***

- Unlike neural network models, where the chemical structures (input) and the binary activities (output) are fed separately to the network, one dataset comprising the structures and the binary activities is directly fed to the H2O system.
- A “target_col” is specified, which is the column to predict; in our case this refers to the ‘binary activity’ column. H2O automatically detects the column named ‘ID’ as the identifier column of the dataset (in our case compounds IDs).
- Evaluation : The final ensemble conformation matrices (TP, FP, TN, FN), ensemble rocs (TPR, FPR, Precision, Recall) and ensemble scores (Accuracy, MCC, AUCPR) of the held-out test sets were retrieved for all the 47 target models in form of .json files and analyzed in R studio 3.5.1.

### ***AutoML with AutoGluon Tabular***

- The same train and test csv files (containing the compounds’ fingerprints and binary activities) utilized for H2O were used for
- A ‘label’ column (containing the binary activities) and an ‘id_column’ (containing compounds ids) are defined.

**Table S2** Neural networks performance metrics for the three target excluded from the analysis due to their poor performance.

| **Target name** | **Hit percent** | **Accuracy** | **Balanced accuracy** | **AUC** | **AUCPR** | **MCC** | **F1** |
| --- | --- | --- | --- | --- | --- | --- | --- |
| Glutamate (PCP) | 0.5 | 0.99 | 0.5 | 0.11 | 0.006 | 0 | 0 |
| Estrogen alpha | 0.5 | 0.98 | 0.5 | 0.34 | 0.007 | 0 | 0 |
| Glycine (Strychnine-insenstive) | 0.17 | 1 | 0 | NaN* | NaN* | 0 | 0 |

*NaN values in AUC and AUCPR due to the absence true positives
