# Supplementary material for: Off-targetP ML: an open source machine learning framework for off-target panel safety assessment of small molecules
Source: J Cheminform. 2022 May 7;14:27. doi: 10.1186/s13321-022-00603-w (PMC9077900; doi:10.1186/s13321-022-00603-w)
Supplement: Supplementary file 2 — Additional file 2: Table S3.A–E Comparison of different performance metrics for the Neural Networks, H2O, AutoGluon, RandomForest and AutoSklearn methods respectively. [file 13321_2022_603_MOESM2_ESM.docx]

**Table S3.A** Comparison of different performance metrics for the neural networks method.

| **Target** | **Gene name** | **BA** | **Accuracy** | **AUC** | **AUCPR** | **MCC** | **F1** |
| --- | --- | --- | --- | --- | --- | --- | --- |
| Adenosine A3 | ADORA3 | 0.7738 | 0.8198 | 0.866 | 0.7191 | 0.5599 | 0.6852 |
| Dopamine D2S | DRD2 | 0.7437 | 0.904 | 0.8585 | 0.5777 | 0.525 | 0.5763 |
| Mu-type opioid | OPRM1 | 0.7064 | 0.8774 | 0.8148 | 0.4305 | 0.4099 | 0.4793 |
| Muscarinic M1 | CHRM1 | 0.8565 | 0.8775 | 0.9215 | 0.9041 | 0.7325 | 0.8229 |
| Seritonin 5-HT2A | HTR2A | 0.7595 | 0.8577 | 0.8086 | 0.6223 | 0.5537 | 0.6383 |
| Adenosine A1 | ADORA1 | 0.699 | 0.9621 | 0.7231 | 0.4156 | 0.4946 | 0.5 |
| NE transporter | SLC6A2 | 0.7555 | 0.8665 | 0.8463 | 0.6598 | 0.524 | 0.6038 |
| Seritonin 5-HT2B | 5-HT2B | 0.7469 | 0.7689 | 0.8112 | 0.7293 | 0.5044 | 0.6844 |
| Dopamine D1 | DRD1 | 0.7121 | 0.9 | 0.8333 | 0.4887 | 0.4575 | 0.5106 |
| Seritonin -5HT1A | HTR1A | 0.7472 | 0.9061 | 0.848 | 0.61 | 0.5419 | 0.5905 |
| Histamine H1 | HRH1 | 0.7714 | 0.9256 | 0.887 | 0.5394 | 0.5545 | 0.5952 |
| Muscarinic M2 | CHRM2 | 0.758 | 0.8422 | 0.8158 | 0.5838 | 0.5053 | 0.6034 |
| Adrenergic β1 | ADRB1 | 0.7894 | 0.9844 | 0.8288 | 0.4963 | 0.666 | 0.6667 |
| Acetylcholineesterase | ACHE | 0.8169 | 0.8976 | 0.9179 | 0.7319 | 0.6582 | 0.7195 |
| 5HT transporter | SLC6A4 | 0.8855 | 0.8996 | 0.9304 | 0.9123 | 0.7845 | 0.8615 |
| GABA A (Cl− channel) | GABRA1(CL-) | 0.73 | 0.8434 | 0.8248 | 0.6171 | 0.5029 | 0.593 |
| Adrenergic α1A | ADRA1A | 0.7769 | 0.871 | 0.8374 | 0.5312 | 0.5263 | 0.6014 |
| Monoamine oxidase | MAOA | 0.5 | 0.9909 | 0.8693 | 0.0324 | 0 | NA |
| Seritonin 5-HT3 | 5-HT3 | 0.6099 | 0.9812 | 0.8348 | 0.3063 | 0.378 | 0.3333 |
| HIV-1 Protease | HIV1-PR | 0.663 | 0.9787 | 0.7721 | 0.2135 | 0.3979 | 0.4 |
| Adrenergic α2A | ADRA2A | 0.7641 | 0.9158 | 0.8638 | 0.5714 | 0.5283 | 0.575 |
| Adrenergic β2 | ADRB2 | 0.6001 | 0.9442 | 0.7322 | 0.3402 | 0.3395 | 0.3125 |
| PPARgamma | PPARG | 0.6077 | 0.8849 | 0.7338 | 0.3488 | 0.2893 | 0.3284 |
| Ca2+ channel (Diltiazem site) | CACNA1C | 0.7562 | 0.8144 | 0.8301 | 0.6722 | 0.5054 | 0.6289 |
| Nicotinic muscle-type | CHRNA1 | 0.6688 | 0.9129 | 0.8118 | 0.4268 | 0.3781 | 0.4211 |
| Prostaglandin F | PTGFR | 0.5357 | 0.9657 | 0.6728 | 0.142 | 0.2626 | 0.1333 |
| Histamine H3 | HRH3 | 0.8114 | 0.914 | 0.872 | 0.5497 | 0.5407 | 0.5789 |
| Xanthine oxidase | XDH | 0.5916 | 0.9617 | 0.532 | 0.1664 | 0.2042 | 0.2222 |
| Glucocorticoid | NR3C1 | 0.6011 | 0.9449 | 0.693 | 0.2328 | 0.2022 | 0.2308 |
| Cyclooxygenase 2 | PTGS2 | 0.7286 | 0.9164 | 0.8211 | 0.4236 | 0.4707 | 0.5161 |
| Choleystokinin 1 | CCKAR | 0.6378 | 0.919 | 0.7658 | 0.2947 | 0.3591 | 0.383 |
| Matrix metallopeptidase 9 | MMP9 | 0.5714 | 0.9832 | 0.9159 | 0.2604 | 0.3748 | 0.25 |
| Angiotensin receptor II | AGTR1 | 0.7486 | 0.9915 | 0.7407 | 0.4108 | 0.5732 | 0.5714 |
| GABA-A (Benzo) | GABRA1(Benzo) | 0.9098 | 0.9422 | 0.9317 | 0.8932 | 0.8147 | 0.8504 |
| Histamine H2 | HRH2 | 0.7662 | 0.939 | 0.8074 | 0.4236 | 0.5324 | 0.5652 |
| Cannabinoid CB1 | CNR1 | 0.6234 | 0.9595 | 0.6756 | 0.2517 | 0.4336 | 0.381 |
| Phosphodiesterase 3B | PDE3B | 0.7076 | 0.9615 | 0.8353 | 0.4679 | 0.4879 | 0.5 |
| ZAP70 Kinase | ZAP70 | 0.625 | 0.9899 | 0.7149 | 0.267 | 0.4975 | 0.4 |
| CDK2 Kinase | CDK2 | 0.6732 | 0.959 | 0.8297 | 0.2693 | 0.4534 | 0.4545 |
| GSK3 beta | GSK3B | 0.669 | 0.946 | 0.906 | 0.1886 | 0.2676 | 0.2857 |
| ABL1 Kinase | ABL1 | 0.9 | 0.9547 | 0.9517 | 0.8795 | 0.8316 | 0.8571 |
| GSK3 alpha | GSK3A | 0.7438 | 0.9261 | 0.8759 | 0.4622 | 0.5195 | 0.5581 |
| Androgen | AR | 0.7017 | 0.9268 | 0.9141 | 0.2539 | 0.3288 | 0.3571 |
| Nicotinic neuronal-type (alpha-BGTX insens.) | CHRNA4 | 0.5 | 0.9947 | 0.9788 | 0.1074 | 0 | NA |
| Angiotensin converting enzyme | ACE2 | 0.5902 | 0.8976 | 0.659 | 0.1177 | 0.1438 | 0.1905 |
| Kappa-type opioid | OPRK1 | 0.7172 | 0.8 | 0.7193 | 0.5601 | 0.4566 | 0.5854 |
| Phosphodiesterase 4D2 | PDE4D2 | 0.5 | 0.9231 | 0.4583 | 0.0662 | 0 | NA |

**Table S3.B** Comparison of different performance metrics for the H20 method.

| **Target** | **Gene name** | **BA** | **Accuracy** | **AUC** | **AUCPR** | **MCC** | **F1** |
| --- | --- | --- | --- | --- | --- | --- | --- |
| Adenosine A3 | ADORA3 | 0.767483 | 0.8127 | 0.8665 | 0.7496 | 0.5381 | 0.6832 |
| Dopamine D2S | DRD2 | 0.742649 | 0.9194 | 0.8719 | 0.6224 | 0.5618 | 0.5913 |
| Mu-type opioid | OPRM1 | 0.733627 | 0.9086 | 0.8282 | 0.5339 | 0.4762 | 0.5397 |
| Muscarinic M1 | CHRM1 | 0.840675 | 0.8715 | 0.9193 | 0.9008 | 0.7197 | 0.8116 |
| Seritonin 5-HT2A | HTR2A | 0.739179 | 0.8536 | 0.821 | 0.6509 | 0.515 | 0.5882 |
| Adenosine A1 | ADORA1 | 0.668573 | 0.9684 | 0.8728 | 0.5085 | 0.555 | 0.4848 |
| NE transporter | SLC6A2 | 0.723421 | 0.8792 | 0.8561 | 0.6411 | 0.5112 | 0.5862 |
| Seritonin 5-HT2B | 5-HT2B | 0.720915 | 0.7646 | 0.8214 | 0.7516 | 0.497 | 0.6911 |
| Dopamine D1 | DRD1 | 0.725538 | 0.9196 | 0.8656 | 0.6084 | 0.5589 | 0.6061 |
| Seritonin -5HT1A | HTR1A | 0.745991 | 0.9127 | 0.8725 | 0.6239 | 0.5665 | 0.6078 |
| Histamine H1 | HRH1 | 0.71565 | 0.9256 | 0.8587 | 0.5282 | 0.5046 | 0.5517 |
| Muscarinic M2 | CHRM2 | 0.77686 | 0.8622 | 0.852 | 0.6186 | 0.5415 | 0.6279 |
| Adrenergic β1 | ADRB1 | 0.745434 | 0.98 | 0.938 | 0.5624 | 0.5719 | 0.5833 |
| Acetylcholineesterase | ACHE | 0.791755 | 0.8753 | 0.8967 | 0.7095 | 0.5873 | 0.67 |
| 5HT transporter | SLC6A4 | 0.851647 | 0.8728 | 0.9271 | 0.9123 | 0.7262 | 0.8242 |
| GABA A (Cl− channel) | GABRA1(CL-) | 0.690171 | 0.83 | 0.8165 | 0.6164 | 0.4526 | 0.5806 |
| Adrenergic α1A | ADRA1A | 0.779284 | 0.8937 | 0.8834 | 0.6548 | 0.5542 | 0.6222 |
| Monoamine oxidase | MAOA | 0.5 | 0.9886 | 0.4966 | 0.0121 | 0.0323 | 0.0274 |
| Seritonin 5-HT3 | 5-HT3 | 0.719824 | 0.9836 | 0.8126 | 0.3744 | 0.5365 | 0.5333 |
| HIV-1 Protease | HIV1-PR | 0.603847 | 0.9834 | 0.7727 | 0.3511 | 0.4675 | 0.3704 |
| Adrenergic α2A | ADRA2A | 0.704396 | 0.9134 | 0.8615 | 0.4791 | 0.4649 | 0.5185 |
| Adrenergic β2 | ADRB2 | 0.575225 | 0.9467 | 0.8025 | 0.3799 | 0.4017 | 0.4348 |
| PPARgamma | PPARG | 0.619073 | 0.8977 | 0.8062 | 0.4093 | 0.3535 | 0.4211 |
| Ca2+ channel (Diltiazem site) | CACNA1C | 0.742123 | 0.8273 | 0.8529 | 0.7049 | 0.5278 | 0.6522 |
| Nicotinic muscle-type | CHRNA1 | 0.674081 | 0.9288 | 0.8341 | 0.4262 | 0.3807 | 0.4035 |
| Prostaglandin F | PTGFR | 0.526125 | 0.9657 | 0.7456 | 0.176 | 0.2626 | 0.2353 |
| Histamine H3 | HRH3 | 0.743566 | 0.9409 | 0.8965 | 0.6242 | 0.5563 | 0.56 |
| Xanthine oxidase | XDH | 0.540169 | 0.9699 | 0.6823 | 0.0853 | 0.1698 | 0.1739 |
| Glucocorticoid | NR3C1 | 0.62956 | 0.9614 | 0.8185 | 0.1707 | 0.315 | 0.3333 |
| Cyclooxygenase 2 | PTGS2 | 0.74914 | 0.9304 | 0.8331 | 0.5226 | 0.4907 | 0.5185 |
| Choleystokinin 1 | CCKAR | 0.572561 | 0.919 | 0.7307 | 0.2256 | 0.2734 | 0.2979 |
| Matrix metallopeptidase 9 | MMP9 | 0.564286 | 0.9776 | 0.8698 | 0.1415 | 0.3157 | 0.25 |
| Angiotensin receptor II | AGTR1 | 0.739943 | 0.9943 | 0.7838 | 0.516 | 0.7051 | 0.6667 |
| GABA-A (Benzo) | GABRA1(Benzo) | 0.867586 | 0.9331 | 0.9369 | 0.8743 | 0.773 | 0.8 |
| Histamine H2 | HRH2 | 0.682537 | 0.9451 | 0.8298 | 0.4735 | 0.4611 | 0.5 |
| Cannabinoid CB1 | CNR1 | 0.613525 | 0.9564 | 0.7695 | 0.2897 | 0.3614 | 0.3404 |
| Phosphodiesterase 3B | PDE3B | 0.775647 | 0.9647 | 0.873 | 0.5267 | 0.5619 | 0.5806 |
| ZAP70 Kinase | ZAP70 | 0.621575 | 0.9899 | 0.6755 | 0.2711 | 0.4975 | 0.4 |
| CDK2 Kinase | CDK2 | 0.635689 | 0.959 | 0.8059 | 0.3073 | 0.3873 | 0.4118 |
| GSK3 beta | GSK3B | 0.551389 | 0.9676 | 0.8843 | 0.1543 | 0.2925 | 0.2449 |
| ABL1 Kinase | ABL1 | 0.868264 | 0.9358 | 0.9588 | 0.8784 | 0.7705 | 0.809 |
| GSK3 alpha | GSK3A | 0.69463 | 0.9222 | 0.8188 | 0.3878 | 0.44 | 0.4889 |
| Androgen | AR | 0.606576 | 0.9553 | 0.8615 | 0.2479 | 0.4088 | 0.4 |
| Nicotinic neuronal-type (alpha-BGTX insens.) | CHRNA4 | 0.5 | 0.9895 | 0.1958 | 0.0065 | 0.0346 | 0.0128 |
| Angiotensin converting enzyme | ACE2 | 0.515032 | 0.9518 | 0.6772 | 0.1608 | 0.233 | 0.2424 |
| Kappa-type opioid | OPRK1 | 0.703463 | 0.8235 | 0.7287 | 0.5794 | 0.4952 | 0.5714 |
| Phosphodiesterase 4D2 | PDE4D2 | 0.479167 | 0.8846 | 0.7812 | 0.225 | 0.3651 | 0.3333 |

**Table S3.C** Comparison of different performance metrics for the AutoGluon method.

| **Target** | **Gene name** | **BA** | **Accuracy** | **AUC** | **AUCPR** | **MCC** | **F1** |
| --- | --- | --- | --- | --- | --- | --- | --- |
| Adenosine A3 | ADORA3 | 0.733279 | 0.8039 | 0.8764 | 0.7701 | 0.5054 | 0.6288 |
| Dopamine D2S | DRD2 | 0.74812 | 0.9117 | 0.8712 | 0.6306 | 0.553 | 0.5965 |
| Mu-type opioid | OPRM1 | 0.727019 | 0.8755 | 0.86 | 0.5677 | 0.4303 | 0.5 |
| Muscarinic M1 | CHRM1 | 0.842233 | 0.8696 | 0.9168 | 0.9002 | 0.7151 | 0.8059 |
| Seritonin 5-HT2A | HTR2A | 0.748071 | 0.8285 | 0.8304 | 0.6631 | 0.4927 | 0.6019 |
| Adenosine A1 | ADORA1 | 0.723961 | 0.9684 | 0.842 | 0.556 | 0.577 | 0.5714 |
| NE transporter | SLC6A2 | 0.722764 | 0.8602 | 0.8719 | 0.658 | 0.4807 | 0.56 |
| Seritonin 5-HT2B | 5-HT2B | 0.718086 | 0.7581 | 0.8378 | 0.7729 | 0.4744 | 0.634 |
| Dopamine D1 | DRD1 | 0.659332 | 0.913 | 0.8674 | 0.6168 | 0.4591 | 0.4595 |
| Seritonin -5HT1A | HTR1A | 0.760862 | 0.917 | 0.9093 | 0.6788 | 0.5888 | 0.6275 |
| Histamine H1 | HRH1 | 0.734524 | 0.9344 | 0.8994 | 0.6389 | 0.5617 | 0.5833 |
| Muscarinic M2 | CHRM2 | 0.757766 | 0.8489 | 0.8431 | 0.5988 | 0.5155 | 0.6092 |
| Adrenergic β1 | ADRB1 | 0.70605 | 0.98 | 0.8459 | 0.4331 | 0.5365 | 0.5263 |
| Acetylcholineesterase | ACHE | 0.82052 | 0.882 | 0.9207 | 0.7712 | 0.6275 | 0.7006 |
| 5HT transporter | SLC6A4 | 0.861859 | 0.8817 | 0.9373 | 0.9215 | 0.7458 | 0.8328 |
| GABA A (Cl− channel) | GABRA1(CL-) | 0.869465 | 0.8967 | 0.9342 | 0.8783 | 0.6931 | 0.7536 |
| Adrenergic α1A | ADRA1A | 0.80408 | 0.8756 | 0.8934 | 0.6996 | 0.5611 | 0.6309 |
| Monoamine oxidase | MAOA | 0.5 | 0.9909 | 0.6789 | 0.0129 | 0 | 0 |
| Seritonin 5-HT3 | 5-HT3 | 0.555556 | 0.9812 | 0.7669 | 0.3158 | 0.3302 | 0.2 |
| HIV-1 Protease | HIV1-PR | 0.553134 | 0.9763 | 0.8284 | 0.2706 | 0.1827 | 0.1667 |
| Adrenergic α2A | ADRA2A | 0.712637 | 0.9233 | 0.8954 | 0.577 | 0.5086 | 0.5373 |
| Adrenergic β2 | ADRB2 | 0.554392 | 0.9315 | 0.7909 | 0.2908 | 0.1741 | 0.1818 |
| PPARgamma | PPARG | 0.626277 | 0.9003 | 0.8299 | 0.4645 | 0.3712 | 0.381 |
| Ca2+ channel (Diltiazem site) | CACNA1C | 0.764595 | 0.8325 | 0.8761 | 0.7298 | 0.5391 | 0.6486 |
| Nicotinic muscle-type | CHRNA1 | 0.668768 | 0.9129 | 0.8732 | 0.5368 | 0.3781 | 0.4211 |
| Prostaglandin F | PTGFR | 0.535714 | 0.9657 | 0.7726 | 0.1845 | 0.2626 | 0.1333 |
| Histamine H3 | HRH3 | 0.769485 | 0.9409 | 0.9081 | 0.6528 | 0.5927 | 0.6207 |
| Xanthine oxidase | XDH | 0.5 | 0.9727 | 0.6537 | 0.0586 | 0 | 0 |
| Glucocorticoid | NR3C1 | 0.56978 | 0.9559 | 0.6831 | 0.2221 | 0.1886 | 0.2 |
| Cyclooxygenase 2 | PTGS2 | 0.682913 | 0.9359 | 0.8705 | 0.5966 | 0.521 | 0.5106 |
| Choleystokinin 1 | CCKAR | 0.563618 | 0.9218 | 0.7658 | 0.3128 | 0.2746 | 0.2222 |
| Matrix metallopeptidase 9 | MMP9 | 0.5 | 0.9804 | 0.7388 | 0.1074 | 0 | 0 |
| Angiotensin receptor II | AGTR1 | 0.747126 | 0.9886 | 0.8693 | 0.3707 | 0.4943 | 0.5 |
| GABA-A (Benzo) | GABRA1(Benzo) | 0.720931 | 0.8054 | 0.8271 | 0.6239 | 0.4339 | 0.5584 |
| Histamine H2 | HRH2 | 0.707555 | 0.9421 | 0.8445 | 0.4541 | 0.4921 | 0.5128 |
| Cannabinoid CB1 | CNR1 | 0.59375 | 0.9595 | 0.8709 | 0.4589 | 0.4241 | 0.3158 |
| Phosphodiesterase 3B | PDE3B | 0.673538 | 0.9615 | 0.9171 | 0.5366 | 0.4546 | 0.4545 |
| ZAP70 Kinase | ZAP70 | 0.621575 | 0.9831 | 0.5771 | 0.2609 | 0.2803 | 0.2857 |
| CDK2 Kinase | CDK2 | 0.601767 | 0.9522 | 0.8144 | 0.3626 | 0.3065 | 0.3 |
| GSK3 beta | GSK3B | 0.615741 | 0.9604 | 0.7912 | 0.2232 | 0.247 | 0.2667 |
| ABL1 Kinase | ABL1 | 0.868213 | 0.9472 | 0.9531 | 0.8914 | 0.7998 | 0.825 |
| GSK3 alpha | GSK3A | 0.643627 | 0.9222 | 0.8794 | 0.4379 | 0.4051 | 0.4118 |
| Androgen | AR | 0.582398 | 0.9472 | 0.846 | 0.2726 | 0.2208 | 0.2353 |
| Nicotinic neuronal-type (alpha-BGTX insens.) | CHRNA4 | 0.5 | 0.9947 | 0.8201 | 0.0143 | 0 | 0 |
| Angiotensin converting enzyme | ACE2 | 0.5 | 0.9518 | 0.7381 | 0.1258 | 0 | 0 |
| Kappa-type opioid | OPRK1 | 0.695527 | 0.8118 | 0.7107 | 0.5636 | 0.4618 | 0.5556 |
| Phosphodiesterase 4D2 | PDE4D2 | 0.458333 | 0.8462 | 0.7917 | 0.1548 | -0.0833 | 0 |

**Table S3.D** Comparison of different performance metrics for the RandomForest method.

| **Target** | **Gene name** | **BA** | **Acc** | **AUC** | **AUCPR** | **MCC** | **F1** |
| --- | --- | --- | --- | --- | --- | --- | --- |
| Adenosine A3 | ADORA3 | 0.7459 | 0.8251 | 0.8684 | 0.7685 | 0.5528 | 0.6502 |
| Dopamine D2S | DRD2 | 0.6924 | 0.8925 | 0.8654 | 0.6205 | 0.4644 | 0.5088 |
| Mu-type opioid | OPRM1 | 0.6658 | 0.885 | 0.8159 | 0.5403 | 0.4346 | 0.4685 |
| Muscarinic M1 | CHRM1 | 0.8343 | 0.8656 | 0.9164 | 0.8981 | 0.7049 | 0.7952 |
| Seritonin 5-HT2A | HTR2A | 0.6949 | 0.8389 | 0.8187 | 0.6466 | 0.474 | 0.5444 |
| Adenosine A1 | ADORA1 | 0.6478 | 0.9663 | 0.8711 | 0.516 | 0.4613 | 0.4286 |
| NE transporter | SLC6A2 | 0.6374 | 0.8493 | 0.8225 | 0.5487 | 0.3545 | 0.4132 |
| Seritonin 5-HT2B | 5-HT2B | 0.7393 | 0.7857 | 0.8453 | 0.7512 | 0.5155 | 0.6574 |
| Dopamine D1 | DRD1 | 0.5932 | 0.8978 | 0.8334 | 0.3529 | 0.2865 | 0.2985 |
| Seritonin -5HT1A | HTR1A | 0.7203 | 0.9105 | 0.8777 | 0.5868 | 0.5376 | 0.5684 |
| Histamine H1 | HRH1 | 0.6558 | 0.9189 | 0.834 | 0.5258 | 0.4117 | 0.4308 |
| Muscarinic M2 | CHRM2 | 0.6933 | 0.8311 | 0.8146 | 0.6167 | 0.4634 | 0.5422 |
| Adrenergic β1 | ADRB1 | 0.6182 | 0.9667 | 0.8783 | 0.366 | 0.2719 | 0.2857 |
| Acetylcholineesterase | ACHE | 0.79 | 0.8929 | 0.922 | 0.7471 | 0.6242 | 0.6842 |
| 5HT transporter | SLC6A4 | 0.8593 | 0.8859 | 0.9439 | 0.9253 | 0.7478 | 0.8271 |
| GABA A (Cl− channel) | GABRA1(CL-) | 0.6939 | 0.8546 | 0.8345 | 0.6247 | 0.4831 | 0.539 |
| Adrenergic α1A | ADRA1A | 0.6862 | 0.8688 | 0.8494 | 0.6309 | 0.4693 | 0.5167 |
| Monoamine oxidase | MAOA | 0.5 | 0.9909 | 0.5588 | 0.0418 | 0 | NA |
| Seritonin 5-HT3 | 5-HT3 | 0.5 | 0.9765 | 0.6803 | 0.1112 | 0 | NA |
| HIV-1 Protease | HIV1-PR | 0.5507 | 0.9715 | 0.8111 | 0.2197 | 0.1354 | 0.1429 |
| Adrenergic α2A | ADRA2A | 0.6429 | 0.9132 | 0.8191 | 0.5102 | 0.389 | 0.4068 |
| Adrenergic β2 | ADRB2 | 0.6987 | 0.9746 | 0.8651 | 0.5781 | 0.5756 | 0.5455 |
| PPARgamma | PPARG | 0.6743 | 0.9205 | 0.7873 | 0.5161 | 0.4579 | 0.4746 |
| Ca2+ channel (Diltiazem site) | CACNA1C | 0.6697 | 0.8062 | 0.757 | 0.6154 | 0.4107 | 0.5033 |
| Nicotinic muscle-type | CHRNA1 | 0.6736 | 0.9367 | 0.8832 | 0.5738 | 0.526 | 0.5 |
| Prostaglandin F | PTGFR | 0.5357 | 0.9656 | 0.7672 | 0.2567 | 0.2626 | 0.1333 |
| Histamine H3 | HRH3 | 0.6653 | 0.9086 | 0.8407 | 0.569 | 0.458 | 0.4688 |
| Xanthine oxidase | XDH | 0.5 | 0.9753 | 0.5994 | 0.0404 | 0 | NA |
| Glucocorticoid | NR3C1 | 0.5305 | 0.9559 | 0.7342 | 0.2095 | 0.1339 | 0.1111 |
| Cyclooxygenase 2 | PTGS2 | 0.6369 | 0.9109 | 0.7706 | 0.4263 | 0.4083 | 0.4074 |
| Choleystokinin 1 | CCKAR | 0.4955 | 0.916 | 0.7238 | 0.1387 | 0.0263 | NA |
| Matrix metallopeptidase 9 | MMP9 | 0.57 | 0.9803 | 0.8817 | 0.2438 | 0.26 | 0.2222 |
| Angiotensin receptor II | AGTR1 | 0.4985 | 0.9716 | 0.7901 | 0.1796 | 0.0086 | NA |
| GABA-A (Benzo) | GABRA1(Benzo) | 0.8462 | 0.9299 | 0.9197 | 0.8582 | 0.768 | 0.8 |
| Histamine H2 | HRH2 | 0.6842 | 0.9482 | 0.7769 | 0.5335 | 0.5329 | 0.5143 |
| Cannabinoid CB1 | CNR1 | 0.6138 | 0.9657 | 0.8242 | 0.4345 | 0.4043 | 0.3529 |
| Phosphodiesterase 3B | PDE3B | 0.6522 | 0.9679 | 0.9379 | 0.5977 | 0.4843 | 0.4444 |
| ZAP70 Kinase | ZAP70 | 0.5983 | 0.9831 | 0.8175 | 0.1835 | 0.3092 | 0.2857 |
| CDK2 Kinase | CDK2 | 0.5 | 0.9589 | 0.781 | 0.2955 | 0 | NA |
| GSK3 beta | GSK3B | 0.6116 | 0.9568 | 0.8904 | 0.4314 | 0.3546 | 0.3333 |
| ABL1 Kinase | ABL1 | 0.8761 | 0.9472 | 0.9703 | 0.9085 | 0.811 | 0.8372 |
| GSK3 alpha | GSK3A | 0.5221 | 0.9219 | 0.827 | 0.3627 | 0.1076 | 0.0909 |
| Androgen | AR | 0.6111 | 0.935 | 0.7393 | 0.2384 | 0.3389 | 0.3333 |
| Nicotinic neuronal-type (alpha-BGTX insens.) | CHRNA4 | 0.5 | 0.9947 | 1 | 1 | 0 | NA |
| Angiotensin converting enzyme | ACE2 | 0.526 | 0.903 | 0.6048 | 0.0858 | 0.0643 | 0.1111 |
| Kappa-type opioid | OPRK1 | 0.5799 | 0.75 | 0.7164 | 0.4543 | 0.1723 | 0.3226 |
| Phosphodiesterase 4D2 | PDE4D2 | 0.6667 | 0.9231 | 1 | 1 | 0.5538 | 0.5 |

**Table S3.E** Comparison of different performance metrics for the Auto-Sklearn method.

| **Target** | **Gene name** | **BA** | **ACC** | **AUC** | **AUCPR** | **MCC** | **F1** |
| --- | --- | --- | --- | --- | --- | --- | --- |
| Adenosine A3 | ADORA3 | 0.7384 | 0.7845 | 0.8566 | 0.7269 | 0.4801 | 0.6325 |
| Dopamine D2S | DRD2 | 0.772 | 0.8829 | 0.8666 | 0.6051 | 0.5032 | 0.5674 |
| Mu-type opioid | OPRM1 | 0.7748 | 0.8833 | 0.8325 | 0.5358 | 0.4972 | 0.5588 |
| Muscarinic M1 | CHRM1 | 0.853 | 0.8775 | 0.9139 | 0.8951 | 0.7325 | 0.8198 |
| Seritonin 5-HT2A | HTR2A | 0.7432 | 0.8264 | 0.8253 | 0.644 | 0.4846 | 0.5951 |
| Adenosine A1 | ADORA1 | 0.7619 | 0.8758 | 0.844 | 0.4096 | 0.3203 | 0.3218 |
| NE transporter | SLC6A2 | 0.7469 | 0.8284 | 0.8558 | 0.6359 | 0.4561 | 0.5574 |
| Seritonin 5-HT2B | 5-HT2B | 0.7272 | 0.7603 | 0.8217 | 0.7538 | 0.4804 | 0.652 |
| Dopamine D1 | DRD1 | 0.7832 | 0.9043 | 0.8681 | 0.5828 | 0.5397 | 0.5926 |
| Seritonin -5HT1A | HTR1A | 0.8019 | 0.8472 | 0.8778 | 0.6052 | 0.4897 | 0.5513 |
| Histamine H1 | HRH1 | 0.7845 | 0.8928 | 0.8712 | 0.5537 | 0.4852 | 0.5333 |
| Muscarinic M2 | CHRM2 | 0.7723 | 0.8511 | 0.8544 | 0.6207 | 0.5332 | 0.6257 |
| Adrenergic β1 | ADRB1 | 0.7814 | 0.9689 | 0.9176 | 0.4745 | 0.4896 | 0.5 |
| Acetylcholineesterase | ACHE | 0.8409 | 0.8575 | 0.9127 | 0.7319 | 0.6095 | 0.6863 |
| 5HT transporter | SLC6A4 | 0.8465 | 0.8728 | 0.9291 | 0.9145 | 0.7276 | 0.8143 |
| GABA A (Cl− channel) | GABRA1(CL-) | 0.863 | 0.9058 | 0.9397 | 0.8762 | 0.7054 | 0.7634 |
| Adrenergic α1A | ADRA1A | 0.7974 | 0.8643 | 0.8832 | 0.6553 | 0.5365 | 0.6104 |
| Monoamine oxidase | MAOA | 0.4943 | 0.9795 | 0.7311 | 0.0197 | -0.0103 | 0 |
| Seritonin 5-HT3 | 5-HT3 | 0.7682 | 0.9718 | 0.8628 | 0.206 | 0.4484 | 0.4545 |
| HIV-1 Protease | HIV1-PR | 0.8055 | 0.9384 | 0.7697 | 0.2031 | 0.3489 | 0.3158 |
| Adrenergic α2A | ADRA2A | 0.7951 | 0.8713 | 0.8597 | 0.4666 | 0.4711 | 0.5185 |
| Adrenergic β2 | ADRB2 | 0.7452 | 0.9239 | 0.7961 | 0.3362 | 0.4293 | 0.4643 |
| PPARgamma | PPARG | 0.6828 | 0.8772 | 0.8354 | 0.4389 | 0.3731 | 0.4419 |
| Ca2+ channel (Diltiazem site) | CACNA1C | 0.7539 | 0.8325 | 0.8597 | 0.7272 | 0.5302 | 0.6369 |
| Nicotinic muscle-type | CHRNA1 | 0.7347 | 0.8259 | 0.8064 | 0.3914 | 0.3292 | 0.3774 |
| Prostaglandin F | PTGFR | 0.6155 | 0.9208 | 0.7757 | 0.1566 | 0.1788 | 0.2105 |
| Histamine H3 | HRH3 | 0.8386 | 0.8602 | 0.8946 | 0.5726 | 0.4806 | 0.5 |
| Xanthine oxidase | XDH | 0.5593 | 0.8989 | 0.693 | 0.0637 | 0.0694 | 0.0976 |
| Glucocorticoid | NR3C1 | 0.6822 | 0.8871 | 0.8167 | 0.1289 | 0.2162 | 0.2264 |
| Cyclooxygenase 2 | PTGS2 | 0.7605 | 0.8719 | 0.8329 | 0.5073 | 0.4153 | 0.4651 |
| Choleystokinin 1 | CCKAR | 0.6553 | 0.7291 | 0.749 | 0.1953 | 0.1912 | 0.2595 |
| Matrix metallopeptidase 9 | MMP9 | 0.56 | 0.9608 | 0.9045 | 0.1011 | 0.1061 | 0.125 |
| Angiotensin receptor II | AGTR1 | 0.704 | 0.9034 | 0.8355 | 0.5127 | 0.1464 | 0.1053 |
| GABA-A (Benzo) | GABRA1(Benzo) | 0.6821 | 0.7919 | 0.8257 | 0.6045 | 0.3715 | 0.5027 |
| Histamine H2 | HRH2 | 0.7666 | 0.9024 | 0.8815 | 0.4568 | 0.4305 | 0.4667 |
| Cannabinoid CB1 | CNR1 | 0.7206 | 0.8629 | 0.7869 | 0.3768 | 0.274 | 0.2903 |
| Phosphodiesterase 3B | PDE3B | 0.7315 | 0.9423 | 0.7453 | 0.4534 | 0.4112 | 0.4375 |
| ZAP70 Kinase | ZAP70 | 0.613 | 0.9662 | 0.744 | 0.2811 | 0.1609 | 0.1667 |
| CDK2 Kinase | CDK2 | 0.7249 | 0.9283 | 0.8187 | 0.4643 | 0.372 | 0.4 |
| GSK3 beta | GSK3B | 0.5861 | 0.9029 | 0.7796 | 0.0793 | 0.1045 | 0.129 |
| ABL1 Kinase | ABL1 | 0.8796 | 0.9358 | 0.9549 | 0.8823 | 0.7663 | 0.8046 |
| GSK3 alpha | GSK3A | 0.7534 | 0.8366 | 0.8224 | 0.4355 | 0.3683 | 0.4167 |
| Androgen | AR | 0.711 | 0.8618 | 0.8545 | 0.2689 | 0.2497 | 0.2609 |
| Nicotinic neuronal-type (alpha-BGTX insens.) | CHRNA4 | 0.4497 | 0.8947 | 0.6508 | 0.0075 | -0.0242 | 0 |
| Angiotensin converting enzyme | ACE2 | 0.7168 | 0.6867 | 0.7603 | 0.1476 | 0.1964 | 0.1875 |
| Kappa-type opioid | OPRK1 | 0.6876 | 0.8 | 0.7049 | 0.5828 | 0.431 | 0.5405 |
| Phosphodiesterase 4D2 | PDE4D2 | 0.4375 | 0.8077 | 0.7708 | 0.1368 | -0.1043 | 0 |
