# Supplementary material for: Off-targetP ML: an open source machine learning framework for off-target panel safety assessment of small molecules
Source: J Cheminform. 2022 May 7;14:27. doi: 10.1186/s13321-022-00603-w (PMC9077900; doi:10.1186/s13321-022-00603-w)
Supplement: Supplementary file 3 — Additional file 3: Fig. S1. Lollipop chart representing the F1 values of each target model for each method. Fig. S2. Box plots comparing the overall F1 values for the high and low hit percent target groups, with respect to (a) machine learning methods and (b) Target protein classes. Fig. S3. PCA of the Roche-Excape combined datasets for thefour targets (a) ABL1 (b) ADRB1 (c) AGTR1 (d) MAOA. Supplementary paragraph on Uniform Manifold Approximation and Projection (UMAP). Fig. S4. Umap of the Roche-Excape combined datasets for the six case studies. Table S4. Comparison of different performance metrics for the Excape models. Table S5. Comparison of different performance metrics for the combined Roche-Excape models. [file 13321_2022_603_MOESM3_ESM.docx]

**Fig.S1** Lollipop chart representing the F1 values of each target model for each method.

*
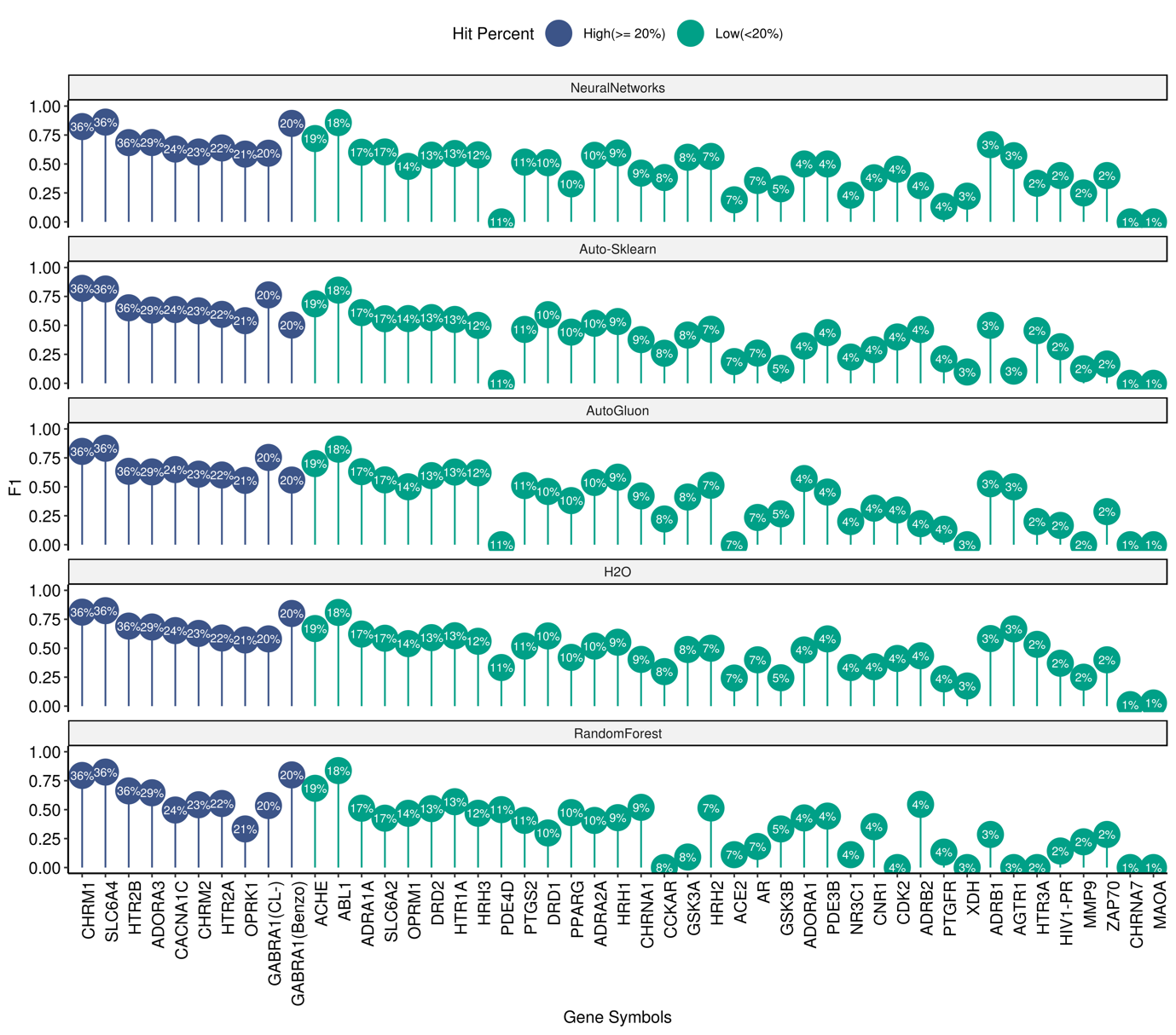
*

Each subplot represents one of the methods used to build the models (Neural networks, Auto-Sklearn, AutoGluon, H2O and Random Forest). The gene names/abbreviations of each target model are represented on the x-axis and the corresponding F1 values on the y axis. Lines are color coded according to the targets’ hit percent category and numerical values of the hit percent are indicated inside the circles.

**Fig.S2** Box plots comparing the overall F1 values for the high and low hit percent target groups, with respect to (a) machine learning methods and (b) Target protein classes

**
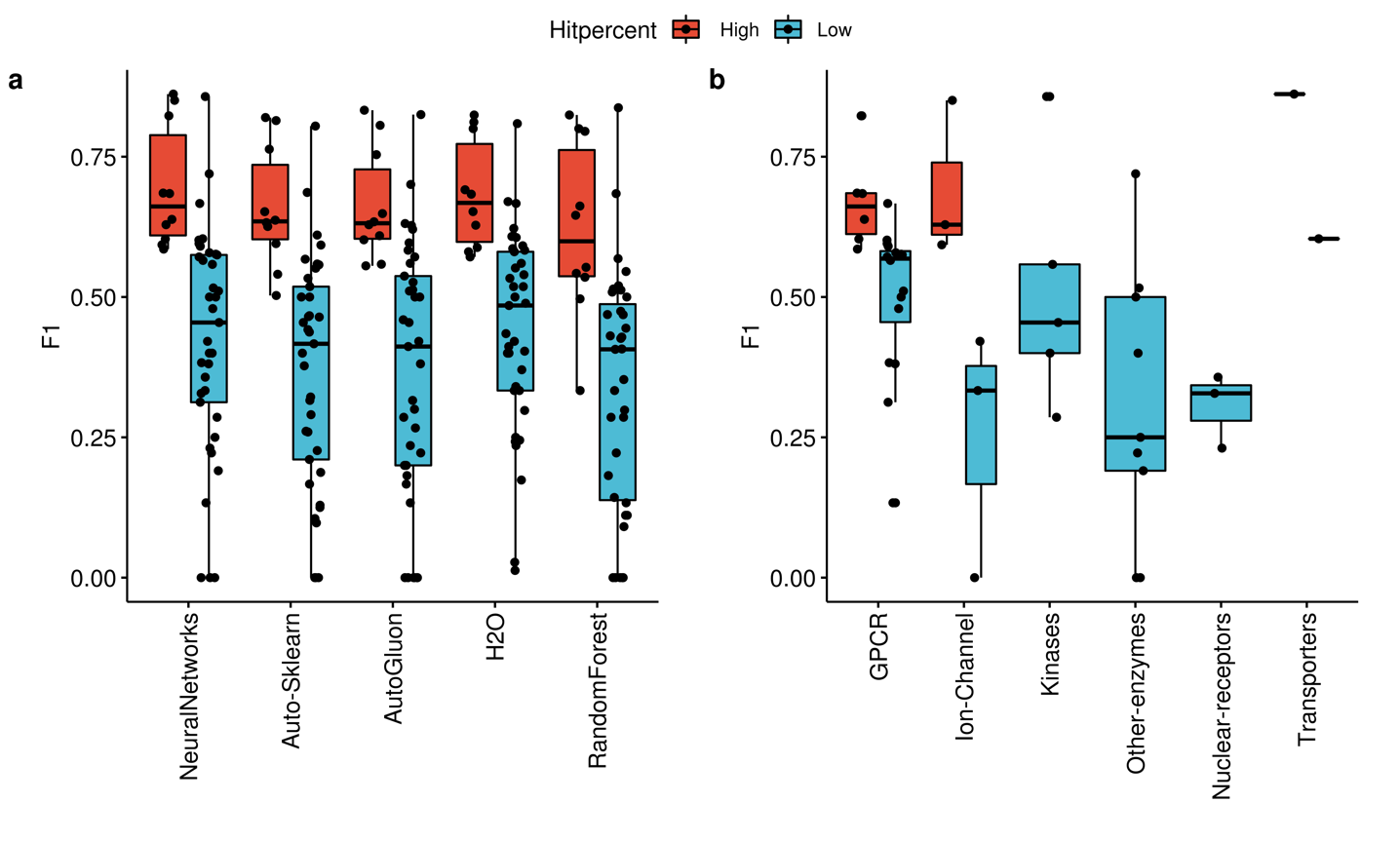
**

1. The five methods are represented on the x axis and the F1 values on the y axis. The red box plots represent the high hit percent target groups and the blue box plots represent the low hit percent target groups. The High hit percent target groups achieve higher F1 irrespective of the method used.
2. The target classes are represented on the x axis and the balanced accuracy values of the neural networks on the y axis. For some target classes, a significant difference is seen in the overall F1 values between the high and low hit percent groups.

**Fig.S3** PCA of the Roche-Excape combined datasets for the four targets: (a) PDE4D and (b) PTGFR.


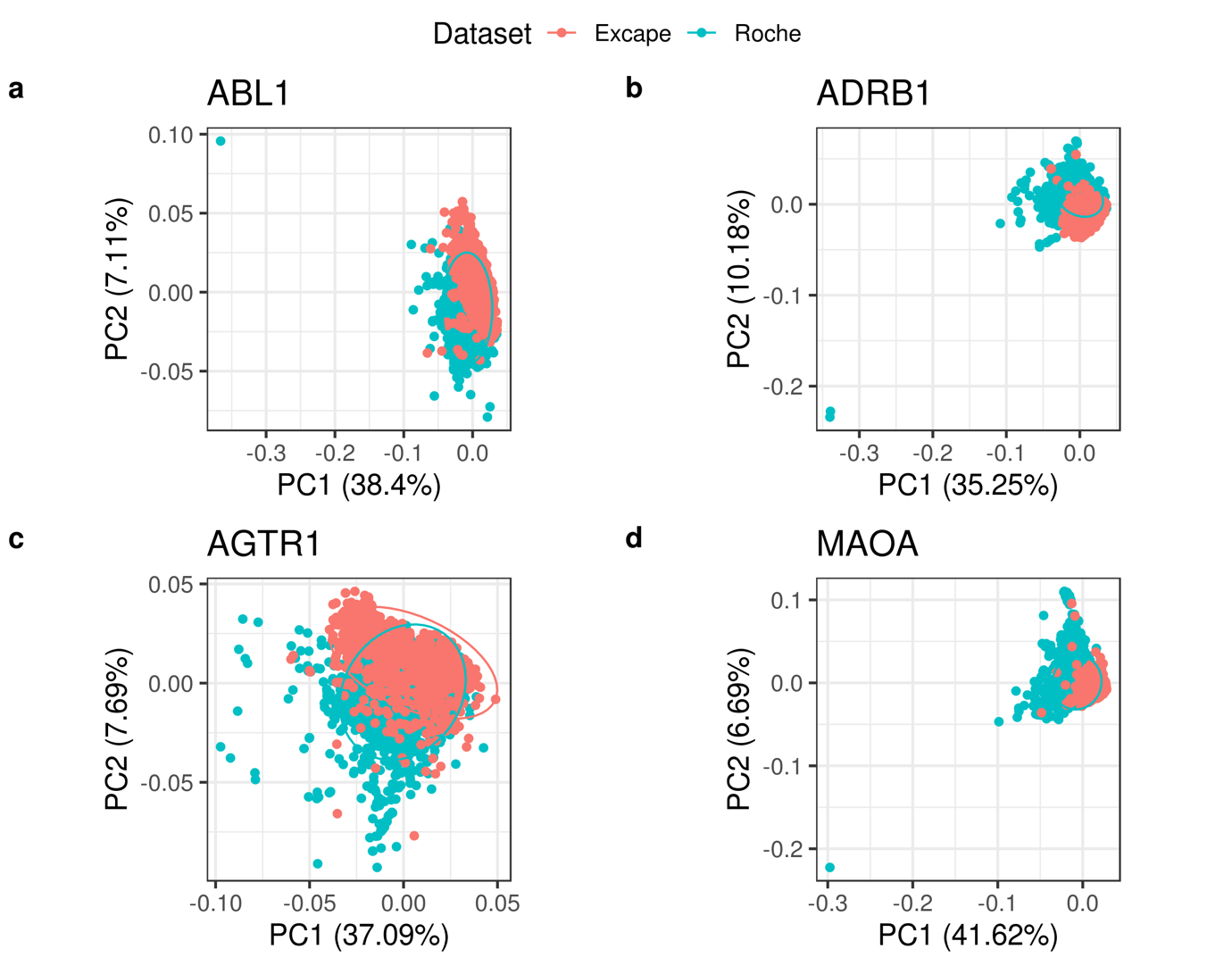


Pink dots represent the Excape compounds, blue dots represent the Roche compounds. The x axis and y axis represent the first two components of the PCA and the percentages indicated represents the variance explained by each component. The ellipses represent the confidence intervals and is implemented through stat_ellipse function in R ggplot2 package.

**Uniform Manifold Approximation and Projection (UMAP)**

Similar to the PCA, the CDK descriptors listed in Additional file 2 (Table S1) were used in the UMAP, where descriptors with zero variance were excluded. The UMAP was conducted using the umap function implemented the umap package version (0.2.6.0) in R version (3.5.1). The default configuration parameters of the umap function were used.

**Figure S4.** Umap of the Roche-Excape combined datasets for the six case studies


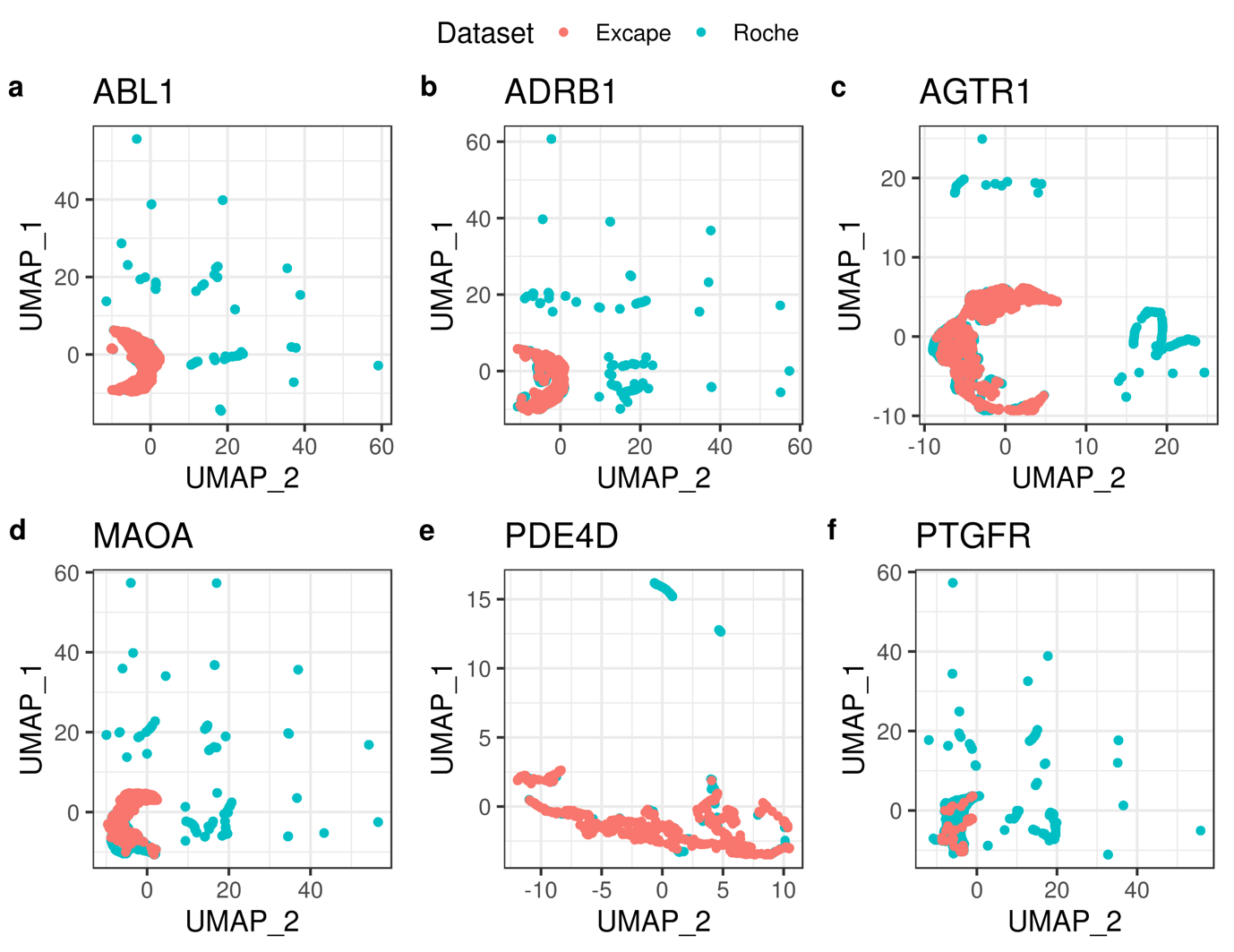


Pink dots represent the Excape compounds, blue dots represent the Roche compounds. The x axis and y axis represent the first two components of the Umap.

**Table S4.** Comparison of different performance metrics for the Excape models.

| Target | BA | Accuracy | MCC | AUC | AUCPR |
| --- | --- | --- | --- | --- | --- |
| ABL1 | 0.684 | 0.926 | 0.361 | 0.83 | 0.987 |
| PDE4D | 0.868 | 0.891 | 0.659 | 0.920 | 0.983 |
| MAOA | 0.839 | 0.840 | 0.679 | 0.881 | 0.874 |
| PTGFR | 0.75 | 0.952 | 0.689 | 0.921 | 0.991 |
| AGTR1 | 0.938 | 0.938 | 0.876 | 0.979 | 0.984 |
| ADRB1 | 0.682 | 0.920 | 0.405 | 0.76875 | 0.972 |

**Table S5.** Comparison of different performance metrics for the combined Roche-Excape models.

| Target | BA | Accuracy | MCC | AUC | AUCPR |
| --- | --- | --- | --- | --- | --- |
| ADRB1 | 0.923 | 0.931 | 0.849 | 0.971 | 0.949 |
| PTGFR | 0.567 | 0.929 | 0.227 | 0.740 | 0.177 |
| AGTR1 | 0.9309 | 0.957 | 0.885 | 0.975 | 0.957 |
| ABL1 | 0.882 | 0.892 | 0.770 | 0.941 | 0.955 |
| MAOA | 0.854 | 0.916 | 0.728 | 0.955 | 0.863 |
| PDE4D | 0.912 | 0.920 | 0.806 | 0.949 | 0.981 |
